# Leveraging Different Distance Functions to Predict Antiviral Peptides with Geometric Deep Learning from ESMFold-Predicted Tertiary Structures

**DOI:** 10.3390/antibiotics15010039

**Published:** 2026-01-01

**Authors:** Greneter Cordoves-Delgado, César R. García-Jacas, Yovani Marrero-Ponce, Sergio A. Aguila, Gabriel Lizama-Uc

**Affiliations:** 1 Centro de Nanociencias y Nanotecnología, Universidad Nacional Autónoma de Mexico, Km. 107 Carretera Tijuana-Ensenada, Ensenada 22860, Baja California, Mexico; 2 Investigador por Mexico, Secretaría de Ciencia, Humanidades, Tecnología e Innovación (Secihti), Ciudad de Mexico 03940, Mexico; 3 Tecnológico Nacional de Mexico, Instituto Tecnológico de Mérida, Unidad de Posgrado e Investigación, Av. Tecnológico, Km. 4.5 S/N, Mérida 97000, Yucatán, Mexico; 4 Facultad de Ingeniería, Universidad Panamericana, Augusto Rodin No. 498, Insurgentes Mixcoac, Benito Juárez, Ciudad de Mexico 03920, Mexico; 5 Grupo de Medicina Molecular y Traslacional (MeM&T), Colegio de Ciencias de la Salud (COCSA), Escuela de Medicina, Edificio de Especialidades Médicas, Universidad San Francisco de Quito (USFQ), Quito 170157, Pichincha, Ecuador

**Keywords:** evolutionary scale modeling, ESM-2, ESMFold, QSAR, antiviral peptides, geometric deep learning, graph deep learning, distance functions

## Abstract

Background: Machine learning models have been shown to be a time-saving and cost-effective tool for peptide-based drug discovery. In this regard, different graph learning-driven frameworks have been introduced to exploit graph representations derived from predicted peptide structures. Such graphs are always derived by applying a Euclidean distance threshold between amino acid pairs, despite the fact that there is no evidence other than intuitive reasoning that supports the Euclidean distance as the most suitable. Objective: In this work, we examined the use of different distance functions to derive graph representations from predicted peptide structures to train deep graph learning-based models to predict antiviral peptides. Methods: To this end, we first analyzed how differently the closeness of the amino acids is characterized by different distance functions. Then, we studied the similarity between the graphs derived with several distance functions, as well as between them and random graphs. Finally, we trained several models with the best graph representations and analyzed how different they are regarding their predictions. Comparisons regarding state-of-the-art models were also performed. Results and Conclusion: We demonstrated that only using Euclidean distance thresholds is not sufficient criterion to build graphs representing structural features of predicted peptide structures, since other distance functions enabled building dissimilar graphs codifying different chemical spaces, which were useful in the construction of better discriminative models.

## 1. Introduction

As of the nineteenth century, viruses were recognized as a type of infectious agent [1,2,3,4]. Scientific developments have allowed large-scale productions of antiviral drugs and vaccines, and their widespread distribution has led to preventing or controlling several viral infections, such as smallpox and poliomyelitis. Nevertheless, viruses continue to be a major cause of various diseases worldwide. Indeed, there are several viruses with relatively few available prophylactics and therapeutics (e.g., HIV, Herpes simplex virus, and Hepatitis virus), which, joined to the emergence and re-emergence of viral epidemics and the ever-increasing reports of antiviral resistance [5,6,7,8], make viral infections constitute a serious threat worldwide. Therefore, researchers constantly work on finding novel molecules that improve the efficacy of antiviral treatments [9], where the use of antiviral peptides (AVPs) has gained a rising attention [10,11,12].

Nowadays, because achieving effective AVPs is a time-consuming, effort-demanding, and expensive process [13], Quantitative Structure Activity Relationship (QSAR) models have been shown to be a time-saving and cost-effective tool for discovering potential AVPs. To date, several QSAR models have been created using non-deep learning-based and deep learning (DL)-based methods [14,15,16,17,18,19,20,21,22,23,24,25]. These models basically perform their predictions using information learned from or calculated on amino acid sequences only. Thus, they do not exploit information derived from the tertiary (3D) structure, such as spatial topology and residue interactions, among others [26]. The lack of 3D information-based models has mainly been due to the low amount of available structural data. However, advances [27,28,29,30,31] in the prediction of the 3D structure of peptides [32] and proteins [33,34] are enabling the use of a great deal of structural data to QSAR practitioners.

Recently, graph learning-driven frameworks [35,36,37] have been developed to exploit graph-based representations derived from predicted peptide structures. The graphs are built by applying a Euclidean metric-based distance threshold between amino acid pairs. Thereby, if the Euclidean distance between the alpha-carbon atoms of every pair of amino acids (graph nodes) is less than or equal to a specified threshold, then an edge is defined. sAMPpred-GAT [35] and deepAMPNet [37] are two of the frameworks created. On the one hand, sAMPpred-GAT uses amino acid–level evolutionary information derived from Position-Specific Scoring Matrices (PSSMs), and receives peptide structures predicted by trRosetta [27] as input. On the other hand, deepAMPNet uses bi-directional long short-term memory (Bi-LSTM)-derived acid-level information, and it is inputted with peptide structures predicted by AlphaFold2 [28]. However, these two frameworks are time- and memory-consuming since they depend on alignment-dependent methods [38]. Thus, they are not proper to be used in the screening of large datasets. To overcome this drawback, we built the esm-AxP-GDL framework [36] (also see Section 5.1), which leverages the ESM-2 and ESMFold alignment-free models to characterize the amino acids and predict the peptide tertiary structures [30], respectively. The models created with esm-AxP-GDL performed consistently better than 20 state-of-the-art (SOTA) models in the prediction of antimicrobial peptides (AMPs).

A principal issue to building good graph deep learning-based models is to properly represent the input data as graphs, such as the graphs representing the geometric information of predicted peptide 3D structures. The concept of the geometrical distance matrix [39] has been commonly used to represent the molecular geometry by calculating the Euclidean metric between amino acid pairs in peptides and proteins, or between atom pairs in small- and medium-sized molecules. The geometrical distance matrix is the basis to derive different 3D molecular/protein descriptors, as well as to derive peptide/protein contact maps [39]. Likely, these are the reasons why both the aforementioned frameworks [35,36,37] and graph learning-driven SOTA applications [40,41,42,43,44,45] only use a Euclidean distance threshold to build graphs from peptide and protein 3D structures, even though there is no evidence other than intuitive reasoning that supports the Euclidean distance as the most suitable.

Inspired by that, Marrero-Ponce et al. [46] introduced the concept of spatial-(dis)similarity matrix as a generalization of the geometrical distance matrix. The spatial-(dis)similarity matrix is calculated using several distance functions (see Table S7 in [39]), including the Euclidean distance. The Euclidean distance is a specific case of the Minkowski definition [47]. Therefore, any function (e.g., Manhattan distance) derived from the Minkowski definition, as well as any function fulfilling the properties of non-negative, symmetry, and reflexivity for all point-pairs (e.g., amino acids) in an N-dimensional space can be used as a distance function. The spatial-(dis)similarity matrix was used in the calculation of 3D molecular descriptors (3D-MDs) [46], where it was demonstrated that distance functions (e.g., Lance–Williams, Clark) other than the Euclidean distance contributed to obtaining 3D-MDs with better modeling ability, and with better ability for discriminating among structurally different molecules [46].

In this manuscript, we follow the concept of the spatial-(dis)similarity matrix and use different distance functions to build graph-based representations of predicted peptide structures to develop graph deep learning-based models. We hypothesize that graph deep learning for modeling AVPs (and peptide/protein activities in general) might benefit from such input graphs, since the use of different distance functions would allow building topologically different graphs aiming to codify different chemical spaces, which would be particularly useful to develop better discriminative models. We implemented these ideas in the esm-AxP-GDL framework [36], and we used a total of 48,927 peptides to demonstrate our hypothesis. The built models were compared to several models reported in the literature to predict AVPs. The esm-AxP-GDL framework is available freely at https://github.com/cicese-biocom/esm-AxP-GDL (accessed on 15 December 2025).

## 2. Results and Discussion

### 2.1. Analysis of the Inter-Amino Acid Distance Distributions

We examined the inter-amino acid distance distributions to figure out how differently the closeness of the amino acids is characterized by different distance functions. We downloaded 34,636 peptide sequences from the StarPep database [48] to carry out this analysis (see Supporting Information (SI) File S1A for the .FASTA file). Section 5.2 details how these sequences were obtained. The tertiary structures of these peptide sequences were predicted with ESMFold [30] (see Section 5.3 for a quality analysis of the predictions). Then, the distance between every pair of amino acids was calculated from the geometric coordinates of the alpha carbon atoms $\left(C_{\alpha }\right)$. A total of 25,688,946 inter-amino acid relationships were analyzed. In addition to the Euclidean distance, we used the Cosine, Bhattacharyya, Canberra, Clark, Lance–Williams, and Soergel distances (see Table S7 in [39] for their mathematical definitions). All these distance functions have been widely used in several bio- and chem-informatics tasks [39,49,50,51].

Table 1 shows measures of dispersion for all distance distributions. Overall, it can be seen that dissimilar distributions are obtained by applying the distance functions accounted for. The Euclidean and Bhattacharyya functions calculate distance values ranging from 0 to infinity. The Euclidean distance-based distribution (also see Figure S1) ranges between 0.72 and 254.06 with an average (and standard deviation) of 20.13 (±13.69), whereas the Bhattacharyya distance-based distribution (see Figure S2) ranges between 0.0912 and 20.36 with an average of 2.74 (±1.62). This shows that more squeezed characterizations of the peptide tertiary structures can be obtained with the Bhattacharyya distance (see Figure S3). This conclusion is also supported by the kurtosis of both distributions. Both are leptokurtic (kurtosis greater than 0), but the Bhattacharyya distance-based distribution has a kurtosis value less than that of the Euclidean distance-based distribution, and thus the former generates much fewer outliers (here, atypical inter-amino acid distances) than the latter (see Figure S4).

**Figure 1:**
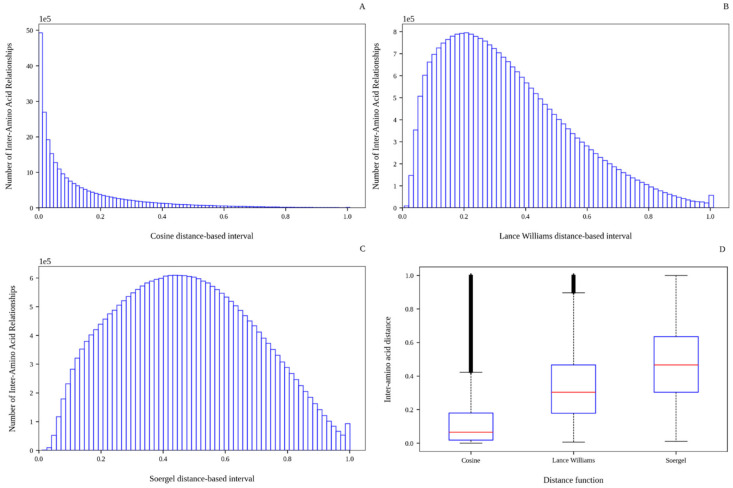
Histograms and boxplots of the inter-amino acid distance distributions obtained with the Cosine (**A**), Lance–Williams (**B**), and Soergel (**C**) distance functions, respectively.

Moreover, the Cosine, Lance–Williams, and Soergel distance functions calculate values ranging from 0 to 1. On the one hand, the Cosine distance-based distribution (Figure 1A) has the greatest excess positive kurtosis, and it is the most positively skewed. In this distribution, 25, 50, and 75% of the distance values are inferior to 0.018, 0.0654, and 0.1799, respectively. Because the Cosine distance is calculated as the difference between 1 and the cosine of the angle between two vectors (here, geometric coordinate vectors), the previous results imply that the angles between 25, 50, and 75% of the inter-amino acid relationships were less than 11, 21, and 35 degrees, respectively. The smaller the angle, the less the distance between two vectors. Therefore, most of the geometric coordinate vectors representing the amino acids in the predicted peptide structures are pointing in roughly the same direction, suggesting that most of the analyzed structures are extended.

On the other hand, the Lance–Williams distance-based distribution (Figure 1B) is moderately positive-skewed (skewness between 0.5 and 1), with an average distance value of 0.34 (±0.20), and with a kurtosis close to 0 that indicates that the distribution tends to be similar to the normal distribution. As for the Soergel distance-based distribution (see Figure 1C), it is fairly symmetrical (skewness ranging from −0.5 to 0.5), platykurtic (kurtosis less than 0), and with an average distance value of 0.47 (±0.22). A platykurtic distribution has thinner tails and presents a lower and broader peak than the normal distribution, resulting in very few or no outliers. Indeed, it can be observed in Figure 1D that the Soergel function did not generate atypical values, while the Lance–Williams function generated a few; the Cosine function generated the greatest number of outliers. All these results indicate that the Soergel and Lance–Williams distance functions yield more stretched distributions (also see Figure 1D), making them more suitable than the Cosine distance function to characterize amino acid pairs that may be spatially close (or contiguous), such as amino acids forming up an alpha helix arrangement (see Figure S5).

Lastly, the Canberra and Clark functions calculate distance values ranging from 0 to p, where p is the number of coefficients in each vector (here, p=3). According to the skewness measure, the Canberra distance-based distribution (see Figure S6) is moderately positive-skewed, whereas the Clark distance-based distribution (see Figure S7) is fairly symmetrical. Both distributions are platykurtic according to their kurtosis values. In addition, it can be drawn that the Clark function allows obtaining more squeezed characterizations of the peptide structures than the Canberra distance (see Figure S8), since the distribution based on Clark has values ranging from 0.0101 to 1.7321 with an average value of 0.71 (±0.36), whereas the distribution based on Canberra presents values ranging from 0.0125 to 3 with an average value of 1.09 (±0.60). A similar statement was drawn between the Euclidean and Bhattacharyya distances.

Overall, the results described above suggest that the use of different distance functions seems to be a suitable way to obtain dissimilar characterizations of the geometrical arrangement of the amino acids that conform up predicted peptide structures. Distance thresholds or intervals derived from dissimilar distance distributions should allow the building of topologically different graphs. The use of such graphs can lead to codifying different chemical spaces of the peptide structures under study, which can be particularly useful in the development of better predictive models. To build these graphs, we decided to use the values corresponding to the 25th, 50th, and 75th percentiles of each of the distributions as distance thresholds. Additional studies to analyze the topology of the graphs built with such distance thresholds are shown below.

### 2.2. Analysis of the Graph Representations Built with Different Distance Functions

A total of 21 graphs per ESMFold-predicted peptide structure were built by applying three threshold values per distance function (a total of seven). The threshold values correspond to the 25th, 50th, and 75th percentiles of the distance distributions analyzed in Table 1. The structures predicted for the 34,636 peptide sequences obtained from StarPep (see Section 5.2 and Section 5.3) were the ones used in this section. Firstly, in order to ascertain if the use of different distance functions allows for obtaining meaningful graphs, we studied for each peptide sequence and its corresponding predicted structure, the pairwise similarity between 30 randomly built graphs and the graph derived from the structure by applying a distance threshold. Thus, a maximum of 1,039,080 similarity coefficients were calculated for each threshold applied per distance function. Section 5.4 describes how the random graphs were generated and how the pairwise similarity is calculated. Data S1 and Table S1 include the raw data and measures of dispersion for those similarity coefficient distributions, respectively.

Overall, the average similarity values regarding the random graphs were from moderate to low, ranging from 0.7 to 0.46. The three lowest average similarity values were less than 0.5, and they correspond to the distributions yielded between the random graphs and the graphs derived with the Cosine, Soergel, and Lance–Williams functions by using 0.018, 0.3035, and 0.1789 as distance thresholds, respectively. Additionally, there are seven distributions presenting average similarity values between 0.5 and 0.6. They correspond to the coefficients calculated between the random graphs and the graphs built both applying the Euclidean distance with the three studied thresholds and applying the Canberra, Clark, Bhattacharyya, and Cosine distances with thresholds equal to 0.6155, 0.4161, 1.5158, and 0.0654, respectively. The other distributions had average similarity values between 0.6 and 0.7. These findings show that random graphs representing geometrical information of the predicted peptide structures cannot be created and, thus, the distance functions and thresholds analyzed are a valid way to derive graphs correctly representing such information.

**Figure 2:**
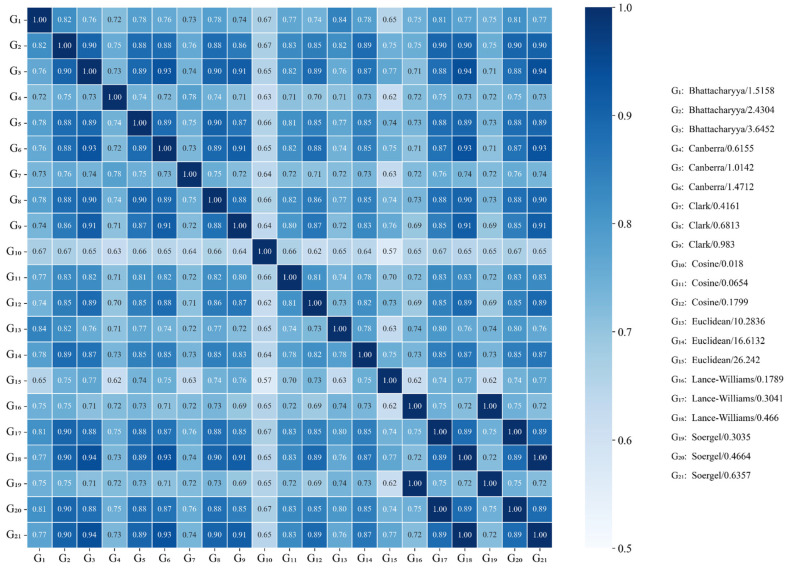
Heat map showing the average similarity between the different graph representations generated for 34,636 predicted peptide structures. For each predicted structure, the similarity coefficient between the graph derived with a specific distance/threshold pair regarding the graph derived with other distance/threshold pair was calculated, and the average of those similarity coefficients is represented in each entry of the heat map.

Moreover, we also examined the similarity between all graph pairs representing the same predicted structure in order to figure out if topologically different graphs can be created by using different distance functions and thresholds. That is, for each predicted structure, we calculated the similarity between the graph derived with a specific distance and threshold regarding the graph derived with another distance and threshold. To this end, we used the 21 graphs built for each of the 34,636 predicted structures. So, a total of 210 similarity relationships were analyzed, and the results are shown in Figure 2 (see also Table S2). Only 32 out of 210 similarity relationships had average similarities from moderate to low at presenting values less than 0.7; whereas 95, 66, and 17 out of 210 distributions had average similarities from moderate to high with values ranging from 0.7 to 0.8 (excluded), from 0.8 to 0.9 (excluded), and from 0.9 to 1 (included), respectively. Therefore, only 32 distance/threshold pairs led to obtaining dissimilar graphs from each other.

Going deeper into the results, it can be seen that the Cosine distance is present in 24 out of the best 32 similarity relationships, mainly when using 0.018 as a distance threshold. That is, this distance function and threshold yielded the most dissimilar graphs compared to those built with the other distance-function/threshold pairs. Regarding the Euclidean distance thresholds, they are present in 10 out of the best 32 similarity relationships, where the threshold of 26.242 was the most representative. However, its use led to mostly building dense graphs, as can be observed in Figure S9. Indeed, more than 50% of the graphs built with the previous threshold had density values greater than 0.9 (maximum value is 1). Euclidean distance thresholds greater than 10 (Angstrom) are rarely used in the literature to build graphs representing protein/peptide tertiary structures, as can be analyzed elsewhere [40,41,42,43,44,45]. The other two Euclidean distance thresholds of 10.2836 and 16.6132 are uniquely present in three out of the best 32 similarity relationships. This suggests that Euclidean distance thresholds are not suitable for building graphs with topologies that differ from those built with other distance functions, since only high thresholds yielding almost complete graphs would allow for achieving good dissimilarities.

Moreover, the threshold values based on the Bhattacharyya and Canberra distance functions are present in four and five out of the best 32 similarity relationships, respectively, whereas the threshold values based on the Clark, Lance–Williams, and Soergel distance functions are present six times each. Unlike the Euclidean distance thresholds, the other distance/threshold pairs allowed building graphs with moderate-to-low density values. Indeed, except the Bhattacharyya threshold of 3.6452, more than 50% of the graphs derived with the non-Euclidean-based distance thresholds presented density values less than 0.8, as can be seen in Figure S9. Overall, these findings support the use of distance functions other than the Euclidean distance to derive dissimilar graph representations of predicted peptide structures, under the assumption that topologically different graphs would contribute to codifying different chemical information. All these previous similarity and density analyses were implemented in the PepProtGraphAnalyzer framework, which is freely available at: https://github.com/cicese-biocom/PepProtGraphAnalyzer (accessed on 15 December 2025).

### 2.3. Analysis of the Models Built with Graphs Derived from Different Distance Functions

**Figure 3:**
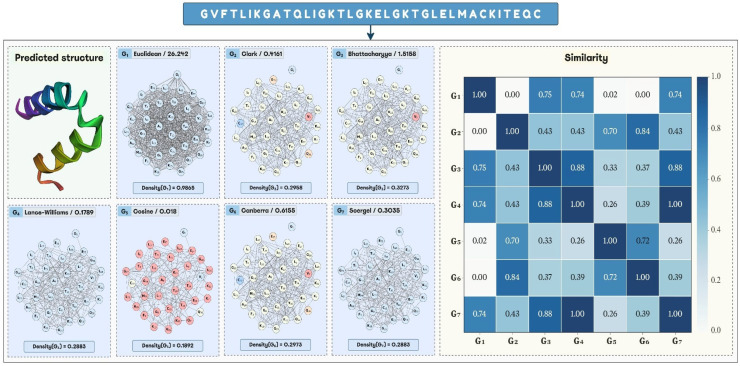
Graph representations built from the ESMFold-predicted structure of a given peptide (StarPep ID: 00743) by applying seven different distance thresholds, namely Euclidean/26.242 (G1), Clark/0.4161 (G2), Bhattacharyya/1.5158 (G3), Lance–Williams/0.1789 (G4), Cosine/0.018 (G5), Canberra/0.6155 (G6), and Soergel/0.3035 (G7). A heat map representing the similarity between each graph pair is also shown.

To carry out this study, we chose the next distance/threshold pairs to build the input graphs to train and test the models: Cosine/0.018, Bhattacharyya/1.5158, Canberra/0.6155, Clark/0.4161, Euclidean/26.242, Lance–Williams/0.1789, and Soergel/0.3035. Examples of graphs built with the previous distance thresholds are shown in Figure 3. Excepting the Euclidean distance threshold of 26.242, the other threshold values belong to the 25th percentile. The Euclidean distance threshold corresponds to the 75th percentile, but it was chosen because the graphs built with it were dissimilar regarding the graphs built with the other distance thresholds mentioned above (see Section 2.2). In this way, it can be analyzed if models created with graphs based on different distance functions, including Euclidean, lead to improving the AVP prediction compared to the use of Euclidean distance-based graphs only, as is performed to date. If better results are achieved by combining those models, then different chemical information is codified with graphs derived from different distance functions and thresholds.

We used the AVPDiscover benchmarking set proposed in [18] for modeling (see Section 5.2). This dataset comprises 4642 training instances (2321 AVPs, 2321 non-AVPs), 1246 validation instances (623 AVPs, 623 non-AVPs), and 12,001 test instances (1230 AVPs, 10,771 non-AVPs). We used the esm-AxP-GDL framework [36] (see Section 5.1) to build all models in this work. The training step was repeated 100 times for each type of input graph, and we selected the model with the highest Matthews Correlation Coefficient $\left(\mathrm{MCC}\right)$ on the validation set $\left({\mathrm{MCC}}_{val}\right)$ each time. In this way, seven pools, each of them comprising 100 models built with the graphs derived from each distance/threshold pair mentioned above, were considered for analysis. File S1B contains the .FASTA files of the AVPDiscover set. Section S1 shows the command lines and parameters used to reproduce the experiments with the esm-AxP-GDL framework.

**Figure 4:**
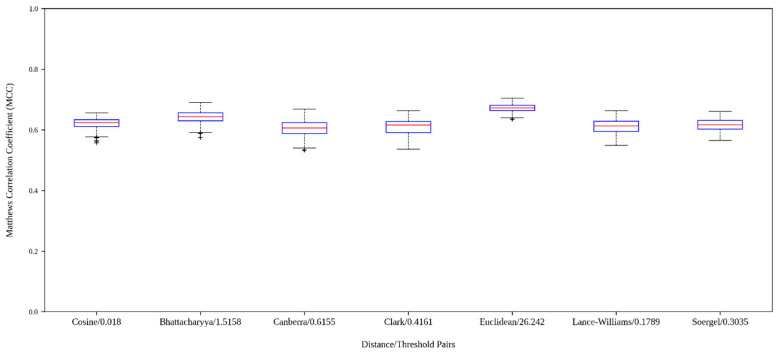
Boxplot graphics corresponding to the Matthews Correlation Coefficients $\left({\mathrm{MCC}}_{val}\right)$ obtained on the AVPDiscover validation test by the models trained with the graphs derived from each distance/threshold pair shown in the figure.

Figure 4 depicts boxplots corresponding to the ${\mathrm{MCC}}_{val}$ values yielded by the built models (see Table S3 for raw data). It can be seen that the values are not scattered from each other in each distribution, which indicates that models with good performance can be consistently trained with the graphs derived from the distance thresholds considered. In this sense, notice that the second quartile of each distribution is superior to 0.6, indicating that at least 50% of all models performed above that value. The best distribution of ${\mathrm{MCC}}_{val}$ values is that of the models based on the graphs built with the Euclidean distance threshold of 26.242, while the second- and third-best distributions correspond to the models created with the graphs built with the Bhattacharyya and Cosine distance thresholds of 1.5158 and 0.018, respectively.

**Figure 5:**
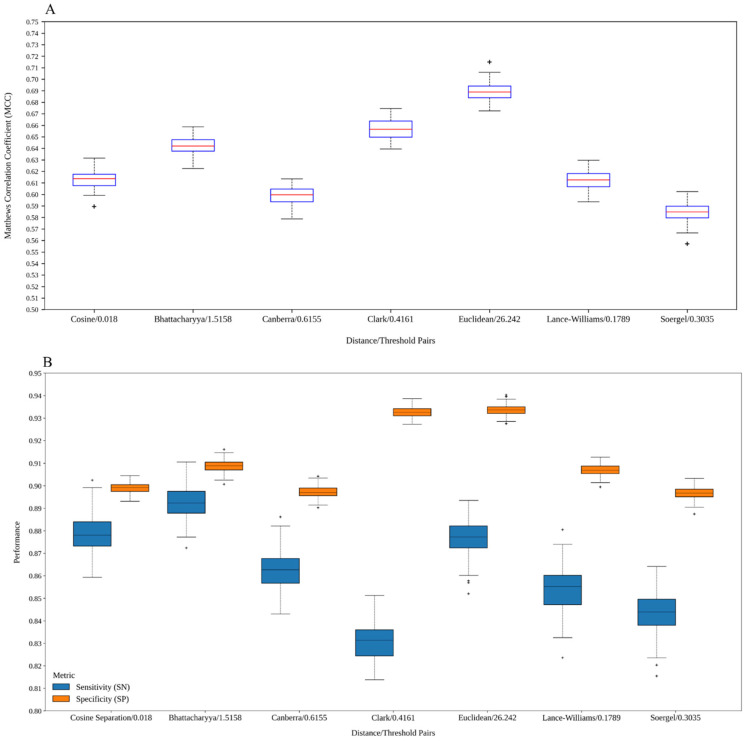
Boxplots corresponding to the MCC, SN, and SP values yielded on the AVPDiscover test set by the best model trained with the graphs derived from the distance/thresholds pairs analyzed in this work. Figure 5A shows the MCC values, whereas the SN and SP values are shown in Figure 5B.

Additionally, we selected the model with the highest ${\mathrm{MCC}}_{val}$ from each pool of 100 models built per distance/threshold pair (see Data S2). These best models were assessed on the test set a total of 100 times to know how stable the predictions are (see Section S2 for the command line). Figure 5 shows boxplots corresponding to the MCC, Sensitivity (SN), and Specificity (SP) values yielded by each best model. In general terms, it can be noted that all distributions are squeezed, indicating that regardless of the random initialization, the models performed similarly among runs. According to the distributions of the ${\mathrm{MCC}}_{test}$ values (see Figure 5A), the model created with the Euclidean distance threshold-derived graphs yielded the best predictions. However, by inspecting the distributions of the ${\mathrm{SN}}_{test}$ values (see Figure 5B), it can be seen that the models built with the graphs derived from the Cosine and Bhattacharyya distance thresholds, respectively, performed better than the model based on the Euclidean distance threshold-derived graphs in the classification of true positives. This supports the hypothesis of this work, since using dissimilar graphs built with distance functions other than the Euclidean distance enabled representing and codifying different chemical spaces to better characterize AVPs.

Moreover, when analyzing the distributions of the ${\mathrm{SP}}_{test}$ values (see Figure 5B), it can be observed that the classification of non-AVPs by the model built with the Clark distance threshold-derived graphs is almost identical to the one yielded by the model developed with the Euclidean distance threshold-derived graphs. Notice also that the other distributions of the ${\mathrm{SP}}_{test}$ values are as good as the two mentioned above. Indeed, the lowest ${\mathrm{SP}}_{test}$ value $\left(=0.8874\right)$ was achieved by the model developed with the Soergel distance threshold-derived graphs, whereas the highest ${\mathrm{SP}}_{test}$ value $\left(=0.9402\right)$ was achieved by the model created with the Euclidean distance threshold-derived graphs, for a range equal to 0.0528. Thus, because the graphs built with different distances led to similarly identifying non-AVPs, it is worth studying if the predictions are complementary or not in order to ascertain if the models codify different chemical spaces (see Section 2.5), which will be ultimately useful in improving the identification of non-AVPs.

### 2.4. Comparative Analysis Regarding Models Reported in the Literature

We made two comparisons: one with regard to several models reported in the literature that were trained and assessed on the AVPDiscover training and test sets (see Table 2A), respectively, and another one with regard to several models reported in the literature that were trained on other datasets but evaluated on the AVPDiscover reduced test set (see Table 2B). The AVPDiscover reduced test set does not contain the training sequences of several models from the literature. This reduced test set comprises 11,460 peptide sequences (689 AVPs, 10,771 non-AVPs), and it was also introduced in [18] (see File S1C for the .FASTA file). For both comparisons, we used the best models built in this work (see Data S2), and we used a seed value equal to 0 to make the predictions (see Data S3). In Table 2A, ProtDCal-AV_RF is the best model based on Random Forest (RF) that was created where the AVPDiscover set was proposed [18]; the ESM-1b feature-based model [52] was the best in a study to evaluate non-handcrafted and handcrafted features in prediction tasks; and the AMPScanner-based model [53] was the best after retraining the AMPScanner deep architecture 30 times [54]. In Table 2B, in addition to the ProtDCal-AV_RF model, we performed comparisons regarding the ClassAMP-SVM [55], iAMP-2L [56], MLAMP [57], AMPfun [58], PEPred-suite [59], iAMPpred [15], Meta-iAVP [16], and Stack-AVP [23] models.

On the one hand, it can be seen in Table 2A that, excepting the model based on the Soergel distance threshold-derived graphs, the other models based on graphs outperformed the best model from the literature $\left({\mathrm{MCC}}_{test}=0.585\right)$ between 2.39 and 16.41% according to the MCC metric. The best model from the literature is that based on RF and ESM-1b features, while the best model in this work is that based on the Euclidean distance threshold-derived graphs $\left({\mathrm{MCC}}_{test}=0.681\right)$, followed by the models developed with the graphs derived from the Clark $\left({\mathrm{MCC}}_{test}=0.6489\right)$ and Bhattacharyya $\left({\mathrm{MCC}}_{test}=0.6471\right)$ distance thresholds, respectively. Notice that the highest ${SN}_{test}$ value was obtained by the best model from the literature $\left({\mathrm{SN}}_{test}=0.921\right)$, being 2.24% better than the highest ${SN}_{test}$ value in this work, which was achieved by the model based on the Bhattacharyya distance threshold-derived graphs $\left({\mathrm{SN}}_{test}=0.9008\right)$. However, according to the ${SP}_{test}$ metric, the best model from the literature $\left({\mathrm{SP}}_{test}=0.8759\right)$ was inferior to the models built in this work between 2.22 $\left({\mathrm{SP}}_{test}=0.8958\right)$ and 5.88% $\left({\mathrm{SP}}_{test}=0.9304\right)$, respectively.

On the other hand, Table 2B shows that all graph-based models were better than all models reported in the literature according to the MCC metric. The model based on the Euclidean distance threshold-derived graphs achieved the highest MCC on the reduced test set $\left({\mathrm{MCC}}_{reduced\text{-}test}=0.5853\right)$, which is 20.46% better than the MCC obtained by the best model from the literature, namely, Stack-AVP [23] $\left({\mathrm{MCC}}_{reduced\text{-}test}=0.4859\right)$. In Table 2B, the graph-based model with the lowest MCC value was created with the graphs derived from the Canberra distance threshold $\left({\mathrm{MCC}}_{reduced\text{-}test}=0.4941\right)$. That MCC value is 1.69% better than the one achieved by the Stack-AVP model. As for the SN metric in Table 2B, the two best results in this work were obtained by the models fed with the graphs built with the Cosine $\left({\mathrm{SN}}_{reduced\text{-}test}=0.8665\right)$ and Bhattacharyya $\left({\mathrm{SN}}_{reduced\text{-}test}=0.8621\right)$ distance thresholds, and both were inferior to the highest SN reported in the literature $\left({\mathrm{SN}}_{reduced\text{-}test}=0.9478\right)$ by 8.58 and 9.04%, respectively.

The best SN value in Table 2B was achieved by Stack-AVP. However, a total of 365 AVP sequences in the AVPDiscover reduced test set are also contained in the Stack-AVP training set (see Table S4). When removing those AVP duplicate sequences (see File S1D for the .FASTA file), it can be observed in Table 2C that Stack-AVP is no longer notably better regarding the SN metric. Indeed, Stack-AVP was only better than the second-best SN value in Table 2C by 1.06%. In consequence, the models based on graphs were much better than Stack-AVP between 12.74 and 35.19% according to the MCC metric (see Table 2C). Moreover, regarding the SP metric in the AVPDiscover reduced test set, it can be seen in Table 2B that the iAMP-2L [56] and MLAMP [57] models achieved almost a perfect performance, but both are biased to predicting non-AVPs since their SN values were less than 0.2. Also notice in Table 2B that all models based on graphs obtained SP values greater than 0.895, which were better than the one achieved by Stack-AVP $\left({\mathrm{SP}}_{reduced\text{-}test}=0.8567\right)$ between 4.58 and 9.17%.

Overall, these outcomes indicate that the use of different distance thresholds other than the Euclidean distance threshold enables building useful graph representations from predicted peptide structures, which leads to building predictive models with consistently good results. However, the models trained with graphs not based on the Euclidean distance threshold were never better than the model fed with the Euclidean distance threshold-derived graphs according to the MCC metric. This would seem to suggest that distance thresholds other than the analyzed Euclidean distance threshold are not useful. However, it can be observed that the models based on the graphs derived from the Cosine and Bhattacharyya distance thresholds yielded the highest SN values, whereas the models based on the graphs derived from the Clark, Bhattacharyya, Lance–Williams, and Soergel distance thresholds performed similarly to the model built with the Euclidean distance threshold-derived graphs according to the SP metric. These observations suggest analyzing if the codified chemical space by these models is different according to the disagreement of their predictions.

### 2.5. Analysis of the Codified Chemical Space According to the Dissimilarity of the Predictions

We assessed the differences in the predictions across all pairs of models trained using graphs constructed at various distance thresholds. To this end, we calculated the disagreement and double-fault measures [60] considering the predictions (see Data S3) performed on the AVPDiscover test set. The disagreement measure is the ratio between the number of predictions on which one model predicts correctly, and the other model predicts incorrectly to the total number of instances. The higher the disagreement value, the higher the chance of obtaining better predictions when combining two models. Moreover, the double-fault measure is the ratio between the number of predictions where both models predict incorrectly to the total number of instances. These two measures were calculated for each class (i.e., AVP, non-AVP), and the results are shown in Table S5. As for the disagreement measure, we reported an adjusted disagreement value for more reliable conclusions. This is equal to the difference between the disagreement per class and the absolute value of the difference between the SN (or SP) values of the analyzed models.

It can be seen in Table S5 that only a few model pairs presented a disagreement greater than 0.1 between their predictions. Above that threshold, the model created with the Euclidean distance threshold-derived graphs presented the least number of disagreements regarding the predictions of the other models. When analyzing the disagreement for the positive class $\left(D_{test}^{+}\right)$, notice that there exists a suitable disagreement (greater than 0.1) between the model trained with the graphs derived from the Cosine distance threshold and the models trained with the graphs derived from the Euclidean $\left(D_{test}^{+}=0.1073\right)$ and Bhattacharyya $\left(D_{test}^{+}=0.1041\right)$ distance thresholds, respectively. As for that metric, the model trained with the graphs created with the Clark distance threshold was also different to the models based on the graphs created with the Soergel $\left(D_{test}^{+}=0.1463\right)$ and Lance–Williams $\left(D_{test}^{+}=0.1187\right)$ distance thresholds, respectively. It is important to highlight that the graphs derived from the Cosine and Euclidean, Cosine and Bhattacharyya, and Clark and Soergel distance thresholds presented low similarity between them (see Section 2.2).

Moreover, when analyzing the disagreement metric for the negative class $\left(D_{test}^{-}\right)$, it can be seen in Table S5 that a greater number of model pairs yielded different predictions between them. The model trained with the graphs derived from the Cosine distance threshold yielded dissimilar predictions to the models built with the graphs created with the Bhattacharyya $\left(D_{test}^{-}=0.1174\right)$, Canberra $\left(D_{test}^{-}=0.1127\right)$ and Soergel $\left(D_{test}^{-}=0.1276\right)$ distance thresholds, respectively. The similarity between the graphs derived from the Cosine distance threshold and the other distance thresholds mentioned above was less than 0.67. That is, those distance/threshold pairs allowed creating dissimilar graphs from predicted peptide structures that led to codifying different chemical information of non-AVPs. Other model pairs also presented suitable $D_{test}^{-}$ values, but the graphs used by them presented moderate similarities (between 0.7 and 0.8) from each other. In this case, the model based on the graphs built with the Bhattacharyya distance threshold performed different predictions to the models based on the graphs derived from the Canberra $\left(D_{test}^{-}=0.117\right)$, Lance–Williams $\left(D_{test}^{-}=0.116\right)$, and Soergel $\left(D_{test}^{-}=0.1118\right)$ distance-based thresholds, respectively. Likewise, the combined models developed with the graphs derived from the Canberra and Soergel $\left(D_{test}^{-}=0.1222\right)$, and Lance–Williams and Soergel $\left(D_{test}^{-}=0.1081\right)$ distance thresholds were different from each other.

All previous results suggest that the recovery rate of true positives (AVPs) or true negatives (non-AVPs) can be improved when combining models performing different predictions. Table 3A and S5 show the performance metrics obtained after combining the model pairs with adjusted disagreement values greater than 0.1. For the model pairs with $D_{test}^{+}>0.1$, if one of them predicts AVP, then the final decision is AVP. On the other hand, for the model pairs with $D_{test}^{-}>0.1$, if one of them predicts non-AVP, then the final decision is non-AVP. As is expected, when combining models with $D_{test}^{+}>0.1$ (see Table S6), the SN values improved regarding the ones obtained by the individual models (see Table 2); however, the MCC values were inferior to the latter. A rather different behavior was achieved by combining the model pairs with $D_{test}^{-}>0.1$ (see Table 3A). In this case, the SP and MCC values were better than the ones obtained by the individual models between 2.49 and 7.19%, and between 3.29 and 11.55%, respectively. This confirms that distance thresholds other than Euclidean distance thresholds are valuable to represent and codify different chemical spaces that can be complemented to achieve better predictive results.

Moreover, despite the fact that the model trained with the graphs derived from the Euclidean distance threshold did not present $D_{test}^{-}$ values greater than 0.1, we combined that model with the models based on the graphs derived from the Cosine and Clark distance thresholds, respectively. Table 3B shows the performance metrics for those fused models. Regarding the MCC metric, the combined models based on the graphs built with the Euclidean and Cosine $\left({\mathrm{MCC}}_{test}=0.7606\right)$, and Euclidean and Clark $\left({\mathrm{MCC}}_{test}=0.7753\right)$ distance thresholds achieved better performance than the best combined model (see Table 3A) that did not use graphs derived from the Euclidean distance threshold $\left({\mathrm{MCC}}_{test}=0.7598\right)$. That best non-Euclidean based model was built with the graphs derived from the Cosine and Bhattacharyya distance thresholds. Finally, we combined the models developed with the graphs derived from the Euclidean, Cosine, and Bhattacharyya distance thresholds. If one of the three models predicts non-AVP, then the prediction is non-AVP. As a result, ${\mathrm{SN}}_{test}$, ${\mathrm{SP}}_{test}$, and ${\mathrm{MCC}}_{test}$ values equal to 0.8016, 0.979, and 0.7858 were achieved, respectively, which are better than the best result reported in Table 3A.

**Figure 6:**
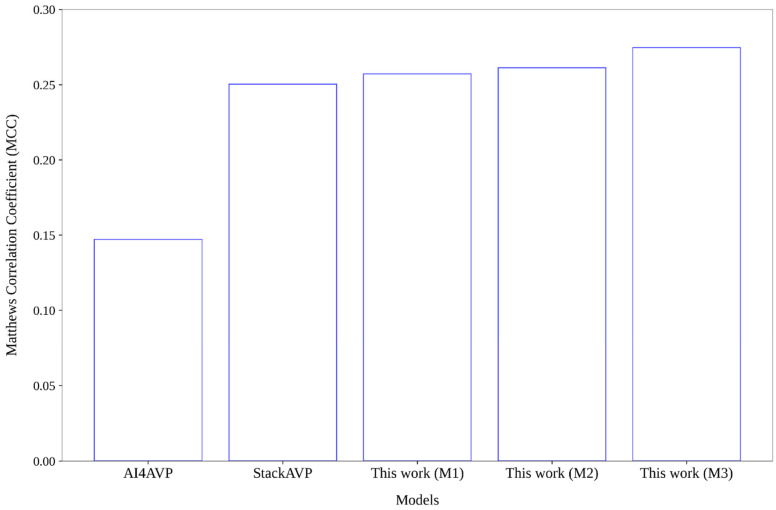
Matthews Correlation Coefficient (MCC) values obtained by the AI4AVP and Stack-AVP models reported in the literature, as well as by the best three graph-based combined models that were developed in this work. The M1, M2, and M3 combined models correspond to the fusing of the output of the individual models fed with the graphs derived from the Euclidean/26.242 and Clark/0.4161 distance thresholds, from the Euclidean/26.242 and Cosine/0.018 distance thresholds, and from the Euclidean/26.242, Cosine/0.018, and Bhattacharyya/1.5158 distance thresholds, respectively.

For more robust conclusions, we built an external set (see Section 5.2) comprising 8903 peptide sequences (273 AVPs, 8630 non-AVPs) with lengths ranging from 10 to 30 amino acids. The best individual (see Data S2) and combined models based on graphs, as well as the Stack-AVP [23] and AI4AVP [61] models from the literature were evaluated on the external set. Figure 6 depicts the MCC values achieved by the AI4AVP and Stack-AVP models, as well as by three combined models that were built by fusing the output of the individual models fed with the graphs derived from the Euclidean and Clark distance thresholds, from the Euclidean and Cosine distance thresholds, and from the Euclidean, Cosine, and Bhattacharyya distance thresholds, respectively. Data S4 contains the predictions of all models and their SN, SP, ACC and MCC metrics are shown in Table S7 in [39]. As can be seen in Figure 6, all graph-based combined models were notably better than AI4AVP and slightly better than Stack-AVP. Stack-AVP was created by stacking 12 different models, and despite that, it was inferior to the graph-based models that only combined two or three individual models. No graph-based individual model was better than Stack-AVP, although all of them were better than AI4AVP. These results demonstrate that Euclidean distance thresholds are not enough to build graphs representing all structural features of predicted peptide structures, since dissimilar graphs can be built by using distance functions other than the Euclidean distance, which enabled developing better discriminative models.

## 3. Conclusions

Herein, we studied the use of different distance functions, including the Euclidean distance, to build graph representations from predicted peptide structures to train deep graph learning-based models to predict AVPs. By using different distance functions, dissimilar characterizations of the geometrical arrangement of the amino acids that conform up predicted peptide structures can be obtained. Thus, different distance thresholds can be applied to build topologically different graphs. Indeed, according to the similarity studies, the graphs derived from the Cosine, Bhattacharyya, Canberra, Clark, Euclidean, Lance–Williams, and Soergel distance functions by using 0.018, 1.5158, 0.6155, 0.4161, 26.242, 0.1789, and 0.3035 as threshold values, respectively, were the most dissimilar ones from each other, presenting pairwise similarity values inferior to 0.6. The Cosine distance threshold allowed obtaining the greatest number of dissimilar graphs with respect to the other ones.

The models trained with the graphs derived from the previous distance thresholds achieved consistently good results, even notably better than all models from the literature. Such models performed different predictions, evidencing that distance thresholds other than Euclidean distance thresholds are valuable to represent and codify different chemical spaces that can be complemented to achieve better predictive results. Overall, we demonstrated that the exclusive use of Euclidean distance thresholds is not enough to derive graphs representing characteristics of predicted peptide structures and, therefore, the use of other distance functions constitutes a prominent approach for building topologically different graphs, which are ultimately useful in obtaining better discriminative models. All the models were created with the esm-AxP-GDL framework, which is available freely at https://github.com/cicese-biocom/esm-AxP-GDL (accessed on 15 December 2025).

## 4. Future Outlooks

Considering the results of this work, we are going to implement a multi-instance architecture that leverages topologically dissimilar graphs built from predicted peptide structures to develop predictive models. In addition, we will also implement Explainable Artificial Intelligence (XAI) approaches to study the underlying relationships behind the predictions of deep models.

## 5. Materials and Methods

### 5.1. Overview of the Esm-AxP-GDL Framework

The esm-AxP-GDL framework [36] was introduced to build alignment-independent models based on graphs built from ESMFold-predicted structures and whose nodes are characterized with evolutionary information derived from the ESM-2 models. A comma-separated value (CSV) file is given as input to this framework. This CSV file contains the identifier, the amino acid sequence, the activity (0 and 1 for negative and positive activities, respectively), and the partition of each peptide. We used the numbers 1, 2 and 3 to represent the training, validation, and test partitions, respectively. The tertiary structure of each peptide sequence in the input file is predicted through the ESMFold model. All predicted structures are saved in Protein Data Bank (PDB) files. These PDB files can be reused to avoid the ESMFold step if the same dataset is used again for another downstream task.

For each predicted peptide structure, the geometrical distance between the α-carbon atoms of every pair of amino acids can be calculated using one of the following seven distance functions: Euclidean, Cosine, Bhattacharyya, Canberra, Clark, Lance–Williams, and Soergel (see Table S7 in [39] for their definitions). If the calculated distance is less than or equal to a given threshold, then an edge is created between those amino acids. In this way, a graph representation is built per predicted structure, where the nodes represent the amino acids, and the edges represent the structural information. Once all graphs are built, those belonging to the training and validation partitions are selected to train a deep graph learning-based model. If no validation data is specified in the input file, then they are extracted from the training data by making a random splitting (80% training, 20% validation). The validation data are used to evaluate each model trained per epoch. A Graph Attention Network (GAT) architecture was implemented to perform the training process. The test set is used when running the test step only. The training step is performed for a specific number of epochs. For every epoch, the cross entropy-based loss value is calculated on the training and validation sets, respectively. The accuracy (ACC), Matthews Correlation Coefficient (MCC), area under the curve (AUC), sensitivity (SN), and specificity (SP) are calculated on the validation and test sets, respectively.

### 5.2. Peptide Datasets

The set of peptide sequences used to analyze the distribution of inter-amino acid distances (see Section 2.1) and perform the similarity study between the graph representations built from each distance function (see Section 2.2) were extracted from the starPep database [48] (denoted as starPepDB). starPepDB is one of the largest bioactive peptide-related repositories reported to date, which comprises 45,120 non-redundant peptide sequences. These sequences were compiled from 40 different public databases (see Table 1 in [62]). From that total of peptide sequences, we first selected those containing between 10 and 100 amino acids. After that, we filtered out the sequences containing non-canonical amino acids. Thereby, a total of 34,636 peptide sequences were finally considered for the analyses mentioned above. File S1A contains the .FASTA file.

Moreover, we used the AVPDiscover benchmarking dataset (see File S1B for the .FASTA files) proposed by Pinacho-Castellanos et al. [18] to perform the modeling tasks in this work (see Section 2.3). A detailed explanation of how this set was created can be found in Section 2.1 in [18]. The AVPDiscover dataset comprises 4642 training sequences (2321 AVPs, 2321 non-AVPs), 1246 validation sequences (623 AVPs, 623 non-AVPs), and 12,001 test sequences (1230 AVPs, 10,771 non-AVPs). The positive sequences (AVPs) of the AVPDiscover set were obtained from the starPepDB database [48,62]. The negative sequences for the training and validation sets were created by Pinacho-Castellanos et al. following several criteria applied in the literature [54,56,63,64]. These authors acquired the negative sequences for the test set from Gabere and Noble [63]. The AVPDiscover reduced test set (see File S1C for the .FASTA file) was also built in [18] and comprises 11,460 sequences (689 AVPs, 10,771 non-AVPs). Except for the Stack-AVP training set, this dataset does not contain duplicates with the training sets of the other models in Table 2B. So, in order to ensure a fair comparison regarding the Stack-AVP model (see Table 2C), we created a new dataset (see File S1D for the .FASTA file) from the AVPDiscover reduced test set by excluding the sequences contained in the Stack-AVP training set (see Table S4). This new dataset comprises 11,095 sequences (324 AVPs, 10,771 non-AVPs).

Lastly, we built an external dataset from an initial pool comprising 2018 AVP sequences downloaded from the dbAMP (v3.0) database [65] and 21,498 negative sequences compiled by Cordoves-Delgado et al. [36] to assess how well different models classify antimicrobial peptides. From that pool, peptide sequences containing non-natural amino acids were filtered out. After that, duplicate sequences with both the entire AVPDiscover benchmarking dataset and the Stack-AVP training dataset were also removed. Then, the sequences with lengths ranging from 10 to 30 amino acids were kept. This criterion was adopted to evaluate the performance of the models in the short-length AVP classification because they are easier and cheaper to synthesize, modify, and optimize than larger AVPs. After these steps, we obtained an external dataset comprising 8903 peptide sequences (273 AVPs, 8630 non-AVPs). File S1E contains the .FASTA file of this dataset.

### 5.3. Perplexity of the ESMFold-Predicted Peptide Structures

We predicted the tertiary structures of the peptide sets used in this work using the ESMFold model, which is available freely at https://github.com/facebookresearch/esm (accessed on 15 December 2025). The perplexity of the peptide sequences was calculated to determine the quality of the predicted structures. For peptide sequences with low perplexity values, the predicted structures are more reliable [30]. Figures S10–S13 shows box-plot graphics corresponding to the perplexity of the 36-layer ESM-2 model on the sets of peptide sequences considered. The boxplot graphics are shown per sequence length interval. The 36-layer ESM-2 model is used by ESMFold to predict the tertiary structure. The perplexity of the 36-layer ESM-2 model ranges between 1 for perfect predictions and 20 for random predictions. It can first be observed that, independently of the length of the sequences, the perplexity distributions tend to 1 and have small interquartile ranges. Additionally, by considering all the perplexity values together, it can be drawn that the average perplexity (standard deviation) of the 36-layer ESM-2 model is 1.56 (±0.24). Therefore, the peptide structures used in this distance study were suitable. The suitability of ESMFold to predict tertiary peptide structures has recently been studied by Yao et al. [65].

### 5.4. Generation of Random Graphs and Similarity Calculation Between Graph Pairs

On the one hand, the random graphs for each sequence were generated using the Erdős–Rényi model [66] implemented in the NetworkX library (*erdos_renyi_graph* function) [67]. In the Erdős–Rényi model, the probability of creating random edges was set to 0.5, while the number of nodes for each random graph was set to be equal to the number of amino acids of each sequence analyzed. On the other hand, in order to calculate the similarity between two graphs (random or not), we first calculated the eigenvalues of the adjacency matrix of each graph and stored them in a vector. In this way, each graph is represented by a vector of eigenvalues. Then, we calculated the cosine similarity between both vectors to obtain the similarity coefficient between the two graphs. The use of eigenvalues (and eigenvectors) of the adjacency matrix (or the Laplacian matrix) to structurally characterize graphs is supported by the spectral graph theory, which has been widely studied to tackle the problem of graph similarity [68,69].

## Figures and Tables

**Table 1:** Dispersion measures for the different inter-amino acid distance distributions.

Distance Functions	Min	Q1 *^a^*	Q2 *^b^*	Average	Std. Dev.	Q3 *^c^*	Max	Skewness	Kurtosis
Euclidean	0.7188	10.2836	16.6132	20.1265	13.6897	26.2420	254.0563	1.5428	3.9371
Bhattacharyya	0.0912	1.5158	2.4304	2.7378	1.6204	3.6452	20.3574	1.0430	1.3395
Cosine	2.8148E-09	0.0180	0.0654	0.1310	0.1644	0.1799	1.0000	1.9995	4.2676
Lance–Williams	0.0057	0.1789	0.3041	0.3398	0.2039	0.4660	1.0000	0.7500	0.0418
Soergel	0.0113	0.3035	0.4664	0.4746	0.2174	0.6357	1.0000	0.1714	−0.7705
Canberra	0.0125	0.6155	1.0142	1.0855	0.5971	1.4712	3.0000	0.5885	−0.1419
Clark	0.0101	0.4161	0.6813	0.7075	0.3612	0.9830	1.7321	0.3124	−0.6337

*^a^*: first quartile or 25th percentile. *^b^*: second quartile, 50th percentile, or median. *^c^*: third quartile, or 75th percentile.

**Table 2:** Performance metrics achieved on the AVPDiscover original (**A**) and reduced (**B**,**C**) test sets both by several models reported in the literature and by the models developed with graphs derived from different distance-based thresholds.

Model	SN	SP	ACC	MCC
**(A) AVPDiscover original test set (12,001 sequences)**				
*This work (*Cosine*/0.018)*	0.8821	0.8972	0.8957	0.6117
*This work (Bhattacharyya/1.5158)*	**0.9008**	0.9086	0.9078	0.6471
*This work (Canberra/0.6155)*	0.8667	0.8958	0.8928	0.5990
*This work (Clark/0.4161)*	0.8341	0.9287	0.9190	0.6489
*This work (Euclidean/26.242)*	0.8764	**0.9304**	**0.9248**	**0.6810**
*This work (Lance–Williams/0.1789)*	0.8496	0.9048	0.8992	0.6056
*This work (Soergel/0.3035)*	0.8382	0.8971	0.8911	0.5828
ProtDCal-AV_RF (see Table 2 in [18])	0.7420	0.8730	0.8600	0.4760
ESM-1b based Random Forest model—see Table 2 in [52]	**0.9210**	0.8680	**0.8730**	**0.5850**
AMPScanner (retrained)—see Table S4 in [53]	0.6293	**0.8759**	0.8560	0.4024
**(B) AVPDiscover reduced test set (11,460 sequences)**				
*This work (*Cosine*/0.018)*	**0.8665**	0.8965	0.8947	0.5088
*This work (Bhattacharyya/1.5158)*	0.8621	0.9099	0.9071	0.5346
*This work (Canberra/0.6155)*	0.8433	0.8959	0.8928	0.4941
*This work (Clark/0.4161)*	0.8113	0.9339	0.9265	0.5641
*This work (Euclidean/26.242)*	0.8389	**0.9353**	**0.9295**	**0.5853**
*This work (Lance–Williams/0.1789)*	0.8389	0.9017	0.8979	0.5031
*This work (Soergel/0.3035)*	0.8331	0.9002	0.8962	0.4966
ProtDCal-AV_RF (see Table 5 in [18])	0.7270	0.8730	0.8640	0.3860
ClassAMP-SVM [55]	0.2510	0.8300	0.7950	0.0510
iAMP-2L [56]	0.1510	**0.9990**	**0.9490**	0.3690
MLAMP [57]	0.0900	**0.9990**	**0.9450**	0.2720
AMPfun [58]	0.2600	0.5430	0.5260	−0.0940
PEPred-suite [59]	0.2120	0.5150	0.4970	−0.1300
iAMPpred [15]	0.8040	0.8570	0.8540	0.4060
Meta-iAVP [16]	0.6650	0.5680	0.5730	0.1110
Stack-AVP [23]	**0.9478**	0.8567	0.8622	**0.4859**
**(C) AVPDiscover reduced test set w/o Stack-AVP training sequences (11,095 sequences)**				
*This work (*Cosine*/0.018)*	0.8673	0.8965	0.8956	0.3878
*This work (Bhattacharyya/1.5158)*	**0.8796**	0.9099	0.9091	0.4197
*This work (Canberra/0.6155)*	0.8642	0.8959	0.8950	0.3853
*This work (Clark/0.4161)*	0.8241	0.9339	0.9307	0.4499
*This work (Euclidean/26.242)*	0.8302	**0.9353**	**0.9322**	**0.4572**
*This work (Lance–Williams/0.1789)*	0.8488	0.9017	0.9001	0.3885
*This work (Soergel/0.3035)*	0.8395	0.9002	0.8984	0.3813
Stack-AVP [23]	**0.8889**	**0.8567**	**0.8577**	**0.3382**

**Table 3:** Performance metrics achieved on the AVPDiscover test set when combining models trained with graphs derived from different distance thresholds. If one of the two models predict non-AVP, then the final decision is non-AVP.

Distance/Threshold Pairs		SN	SP	ACC	MCC
**(A) Euclidean distance threshold-derived graph-free combined models**					
Cosine/0.018	Bhattacharyya/1.5158	0.8301	0.9673	0.9533	0.7598
	Canberra/0.6155	0.8252	0.9536	0.9404	0.7112
	Soergel/0.3035	0.7976	0.9610	0.9443	0.7165
Bhattacharyya/1.5158	Canberra/0.6155	0.8236	0.9671	0.9524	0.7549
	Lance–Williams/0.1789	0.8098	0.9668	0.9507	0.7444
	Soergel/0.3035	0.8000	0.9645	0.9477	0.7301
Canberra/0.6155	Soergel/0.3035	0.7951	0.9582	0.9415	0.7057
Lance–Williams/0.1789	Soergel/0.3035	0.7886	0.9589	0.9414	0.7034
**(B) Euclidean distance threshold-derived graph-dependent combined models**					
Euclidean/26.242	Cosine/0.018	0.8228	0.9689	0.9539	0.7606
	Clark/0.4161	0.7902	0.9783	0.9590	0.7753

## Data Availability

The original contributions presented in this study are included in the article/supplementary material. Software Availability: https://github.com/cicese-biocom/esm-AxP-GDL (accessed on 15 December 2025).

## Supplementary Materials

The following supporting information can be downloaded at: https://www.mdpi.com/article/10.3390/antibiotics15010039/s1.
