# Supplementary material for: Leveraging Different Distance Functions to Predict Antiviral Peptides with Geometric Deep Learning from ESMFold-Predicted Tertiary Structures
Source: Antibiotics (Basel). 2026 Jan 1;15(1):39. doi: 10.3390/antibiotics15010039 (PMC12837384; doi:10.3390/antibiotics15010039)
Supplement: Supplementary file 1 [file antibiotics-15-00039-s001.zip › antibiotics-3964070-supplementary.pdf]

Supporting Information

Leveraging Different Distance Functions to Predict Antiviral Peptides with Geometric Deep Learning from ESMFold-Predicted Tertiary Structures

Greneter Cordoves-Delgado,[1] César R. García-Jacas (✉),[2-3] Yovani Marrero-Ponce (✉),[4-5] Sergio A. Aguila-Puentes,[1] and Gabriel Lizama-Uc.[3]

[1]*Centro de Nanociencias y Nanotecnologia, Universidad Nacional Autonoma de Mexico, Km. 107 Carretera Tijuana-Ensenada, Ensenada, Baja California, Mexico, C.P. 22860*
[2]*Investigador por México, Secretaría de Ciencia, Humanidades, Tecnología e Innovación (Secihti), Ciudad de México, México.*
[3]*Tecnológico Nacional de México, Instituto Tecnológico de Mérida, Unidad de Posgrado e Investigación, Av. Tecnológico, Km. 4.5 S/N, Mérida, 97000, México.*
[4]*Facultad de Ingeniería, Universidad Panamericana, Augusto Rodin No. 498, Insurgentes Mixcoac, Benito Juárez, Ciudad de México, 03920, México.*
[5]*Universidad San Francisco de Quito (USFQ), Grupo de Medicina Molecular y Traslacional (MeM&T), Colegio de Ciencias de la Salud (COCSA), Escuela de Medicina, Edificio de Especialidades Médicas, Quito, 170157, Pichincha, Ecuador.*

Corresponding authors (✉):

César R. García-Jacas

cesarrjacas1985@gmail.com

Yovani Marrero-Ponce

ymarrero77@yahoo.es

Contents

Leveraging Different Distance Functions to predict Antiviral Peptides with Geometric Deep Learning from ESMFold-predicted tertiary structures 1

Figure S1. Histogram of the inter-amino acid distance distributions calculated with the Euclidean distance function. 4

Figure S2. Histogram of the inter-amino acid distance distributions calculated with the Bhattacharyya distance function. 4

Figure S3. Boxplots corresponding to the inter-amino acid distances calculated with the Euclidean and Bhattacharyya functions on the predicted tertiary structure of the peptide shown below. It can be noted that more squeezed characterizations of the peptide tertiary structures can be obtained with the Bhattacharyya distance. 5

Figure S4. Boxplots of the inter-amino acid distance distributions calculated with the Bhattacharyya and Euclidean distance functions. It can be noted that thus the former generates much fewer outliers (here, atypical inter amino acid distances) than the latter. 6

Figure S5. Boxplots corresponding to the inter-amino acid distances calculated with the Cosine, Lance-Williams, and Soergel distance functions on the predicted tertiary structure of the peptide shown below. It can be noted that the Soergel and Lance-Williams distances produced more stretched distributions, making them more suitable than the Cosine distance to characterize amino acid-pairs that may be spatially close (or contiguous) 7

Figure S6. Histogram of the inter-amino acid distance distributions calculated with the Canberra distance function. 8

Figure S7. Histogram of the inter-amino acid distance distributions calculated with the Clark distance function. 8

Figure S8. Boxplots of the inter-amino acid distance distributions calculated with the Canberra and Clark distance functions. 9

Figure S9. Boxplots of the density values corresponding to the graphs built with each of the distance thresholds studied in this work. 9

Figure S10. Boxplots of the perplexity values calculated for the peptide sequences extracted from the starPep database. 10

Figure S11. Boxplots of the perplexity values calculated for the sequences belonging to the AVPDiscover training set. 11

Figure S12. Boxplots of the perplexity values calculated for the sequences belonging to the AVPDiscover validation set. 11

Figure S13. Boxplots of the perplexity values calculated for the sequences belonging to the AVPDiscover test set. 12

Files S1. FASTA files of the datasets used in this work: 12

Data S1. Raw data of the cosine similarity coefficients calculated between each of the 30 graphs randomly built for each peptide sequence with regard to the graph derived from each predicted structure when applying the 21 distance-based thresholds considered: 12

Data S2. Best model developed with the graphs derived from each of the following distance/threshold pairs: Cosine/0.018, Bhattacharyya/1.5158, Canberra/0.6155, Clark/0.4161, Euclidean/26.242, Lance-Williams/0.1789, and Soergel/0.3035. 12

Data S3. Predictions on the AVPDiscover test set and AVPDiscover reduced test set performed by the best model built with the graphs derived from each of the following distance/threshold pairs: Cosine/0.018, Bhattacharyya/1.5158, Canberra/0.6155, Clark/0.4161, Euclidean/26.242, Lance-Williams/0.1789, and Soergel/0.3035. 12

Data S4. Predictions on the external set performed by the best model built with the graphs derived from each of the following distance/threshold pairs: Cosine/0.018, Bhattacharyya/1.5158, Canberra/0.6155, Clark/0.4161, Euclidean/26.242, Lance-Williams/0.1789, and Soergel/0.3035. 12

Section S1. Command line used to train the models. This command line was repeated 100-times for each distance/threshold pair. 13

Section S2. Command line used to run the test model with a given model. 13

Table S1. Measures of dispersion of the 21 similarity distributions obtained by calculating the cosine similarity between each of the 30 graphs randomly built for each peptide sequence with regard to the graph derived from each predicted structure when applying the 21 distance-based thresholds considered. 15

Table S2. Measures of dispersion of the similarity distributions obtained by calculating the cosine similarity coefficient between the graph derived with a specific distance and threshold regarding the graph derived with another distance and threshold for each peptide sequence of the StarPep-derived dataset........ 16

Table S3. Mathews Correlation Coefficients obtained on the AVPDiscover validation set by the 100 models trained on the AVPDiscover training set by using graphs derived from the following distance/threshold pairs: Cosine/0.018, Bhattacharyya/1.5158, Canberra/0.6155, Clark/0.4161, Euclidean/26.242, Lance-Williams/0.1789, and Soergel/0.3035. The measures of dispersion are shown at the bottom of this table.23

Table S4. Peptide sequences that are contained both in the AVPDiscover reduced test set and in the Stack-AVP training set........ 27

Table S5. Disagreement and double-fault measures calculated between the predictions of the model pairs that were developed from the analyzed distance/threshold pairs. Between parentheses, the adjusted disagreement values are reported. 39

Table S6. Performance metrics achieved on the AVPDiscover test set when combining models trained with graphs derived from different distance thresholds, and whose adjusted disagreement values for the positive class were greater than 0.1. If one of the two models predict AVP, then the final decision is AVP. 40

Figure S1. Histogram of the inter-amino acid distance distributions calculated with the Euclidean distance function.

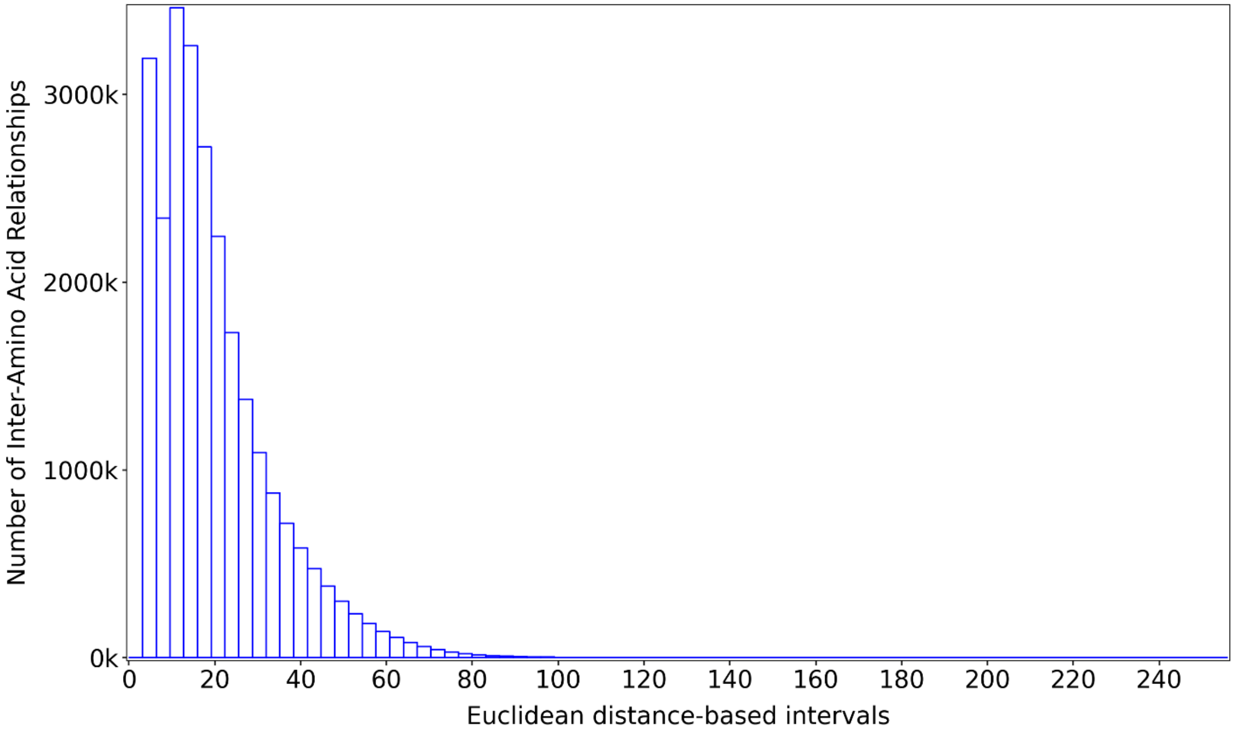

Figure S2. Histogram of the inter-amino acid distance distributions calculated with the Bhattacharyya distance function.

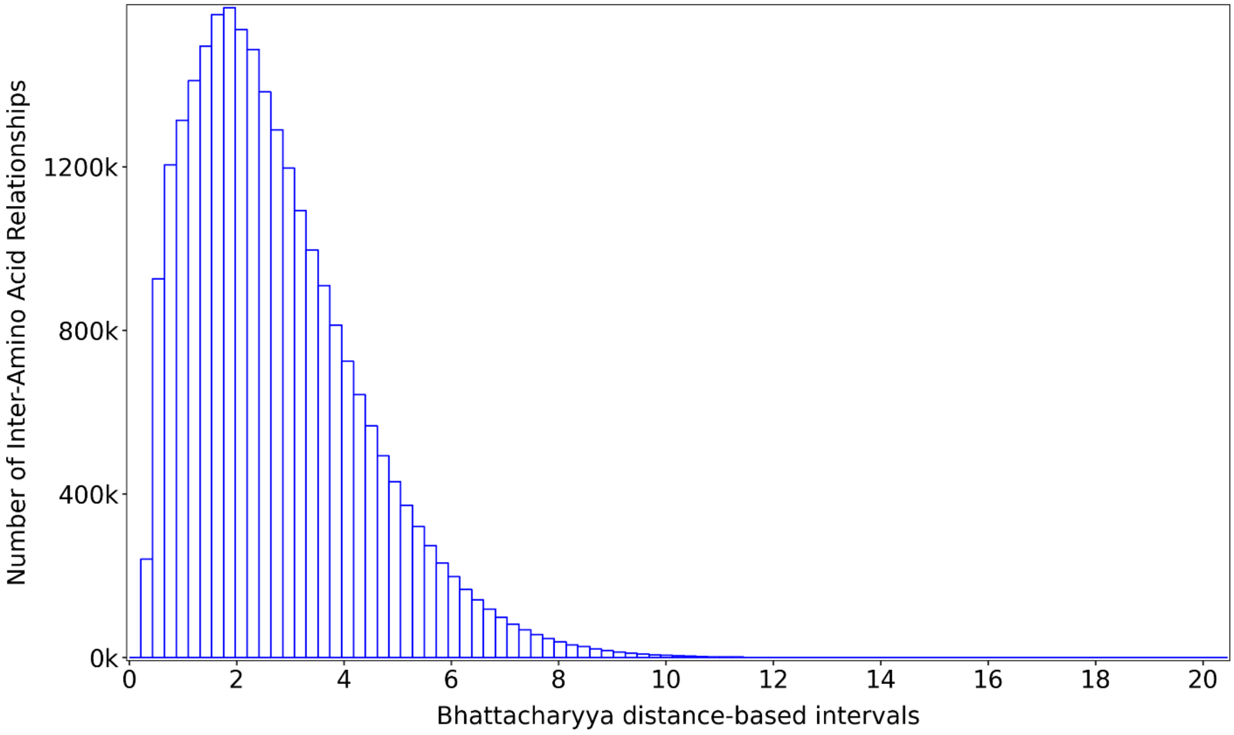

Figure S3. Boxplots corresponding to the inter-amino acid distances calculated with the Euclidean and Bhattacharyya functions on the predicted tertiary structure of the peptide shown below. It can be noted that more squeezed characterizations of the peptide tertiary structures can be obtained with the Bhattacharyya distance.

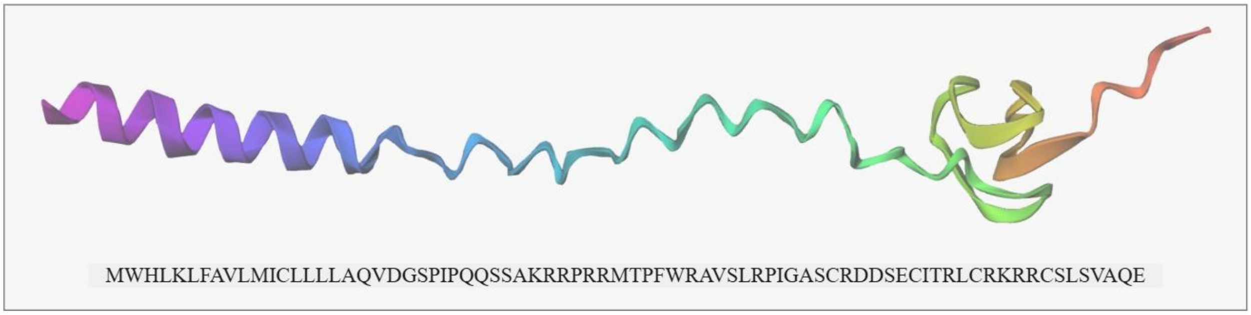

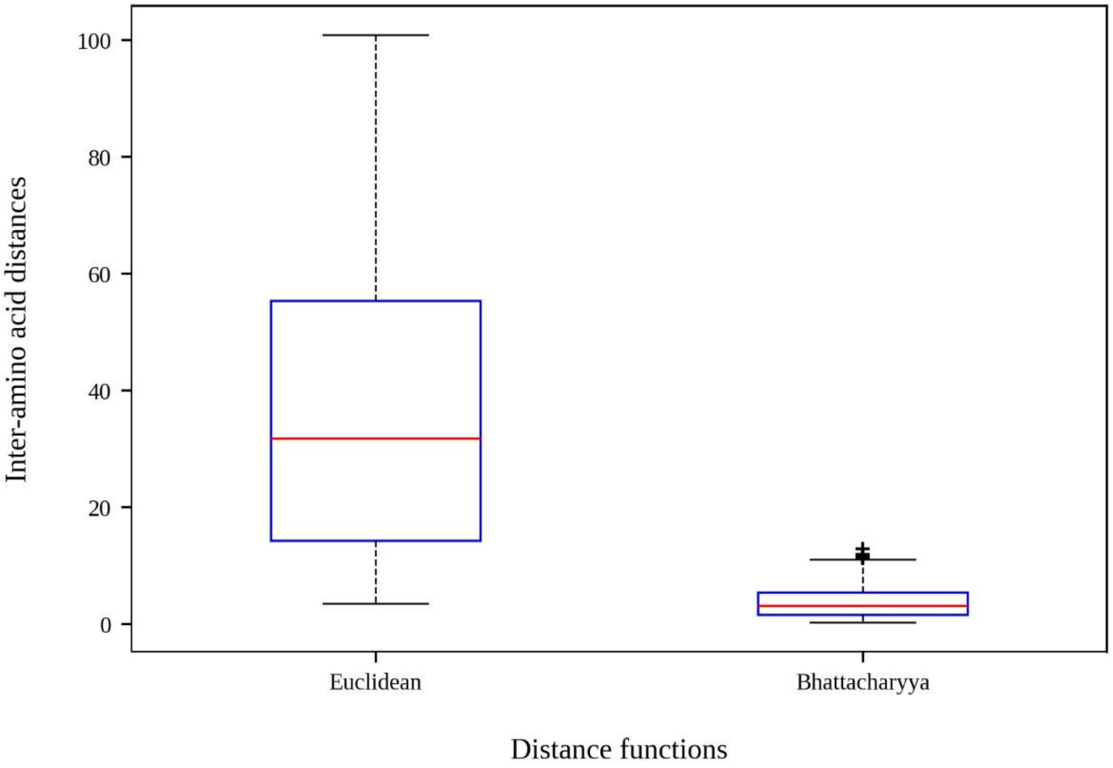

Figure S4. Boxplots of the inter-amino acid distance distributions calculated with the Bhattacharyya and Euclidean distance functions. It can be noted that thus the former generates much fewer outliers (here, atypical inter amino acid distances) than the latter.

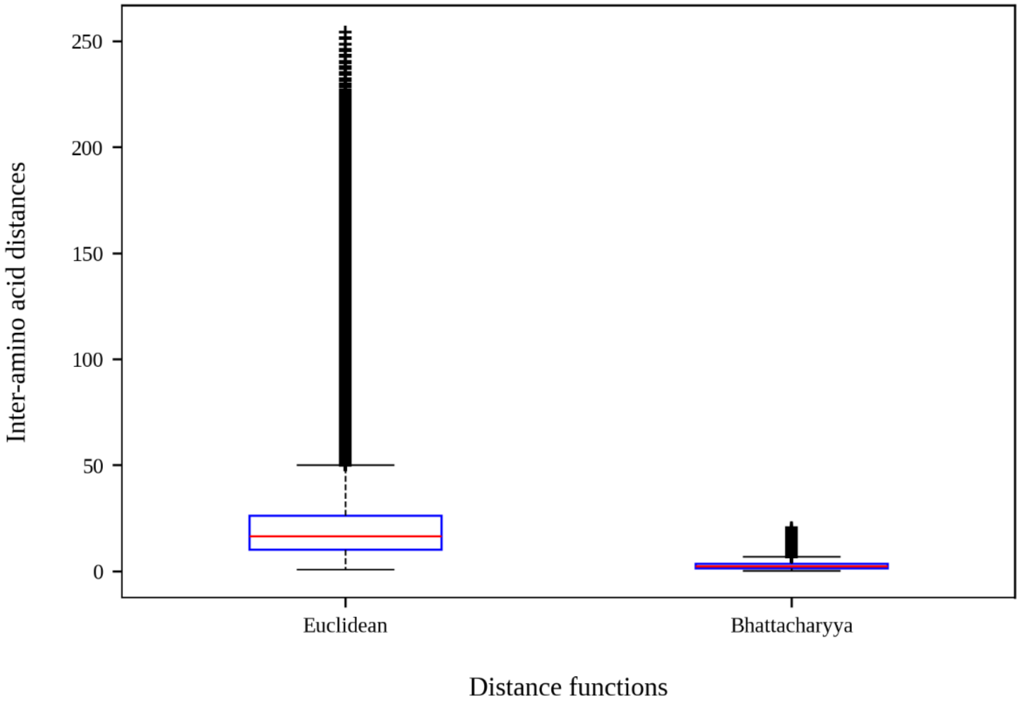

Figure S5. Boxplots corresponding to the inter-amino acid distances calculated with the Cosine, Lance-Williams, and Soergel distance functions on the predicted tertiary structure of the peptide shown below. It can be noted that the Soergel and Lance-Williams distances produced more stretched distributions, making them more suitable than the Cosine distance to characterize amino acid-pairs that may be spatially close (or contiguous).

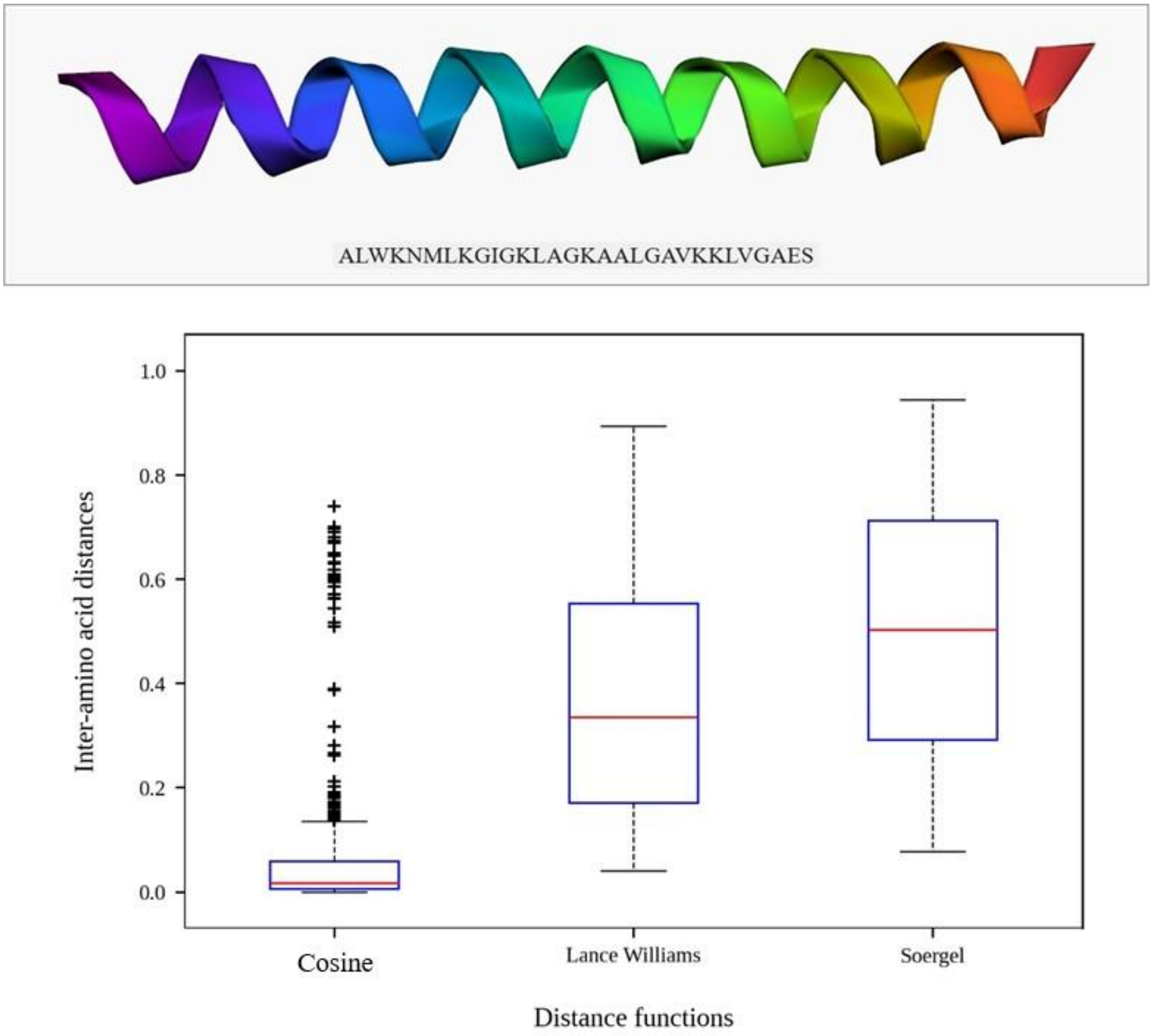

Figure S6. Histogram of the inter-amino acid distance distributions calculated with the Canberra distance function.

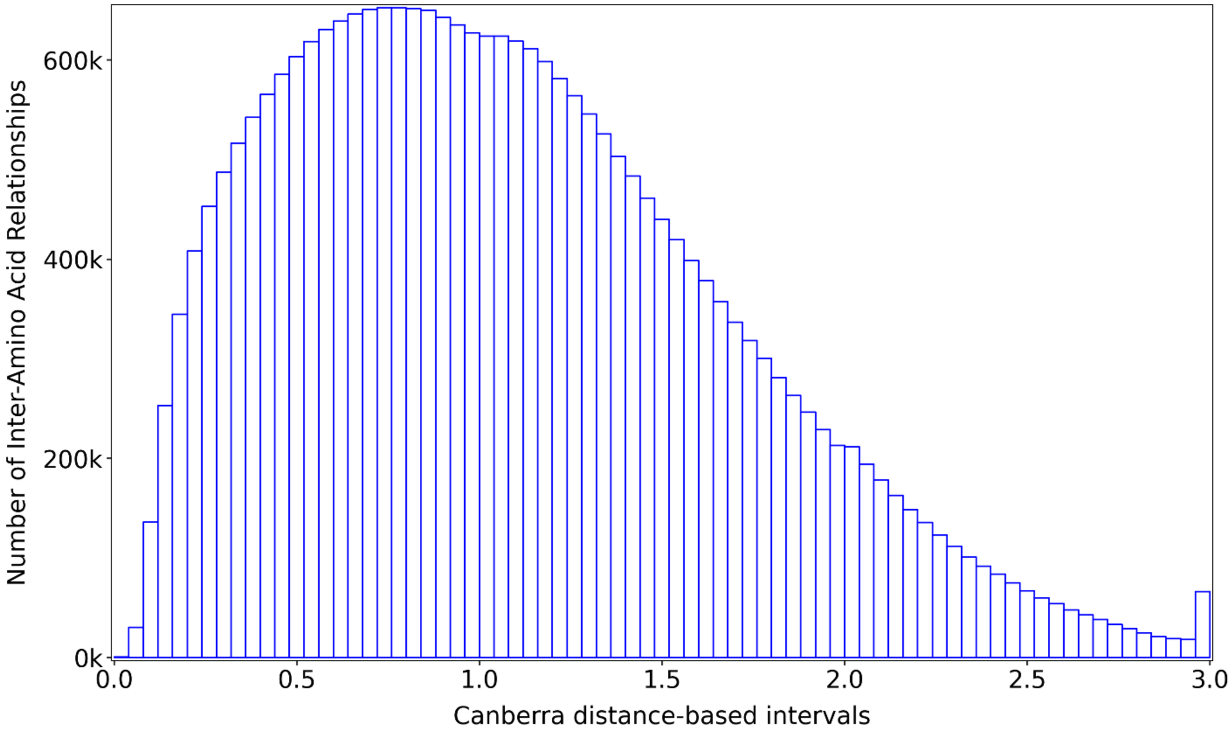

Figure S7. Histogram of the inter-amino acid distance distributions calculated with the Clark distance function.

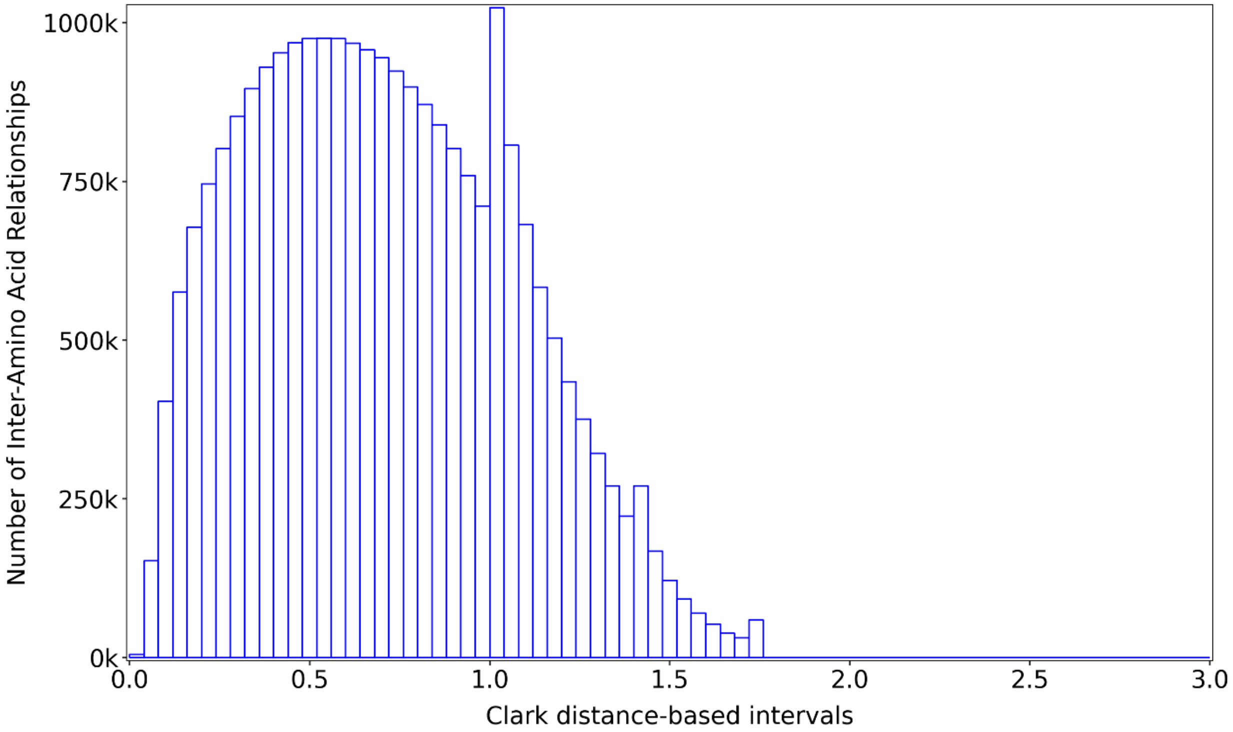

Figure S8. Boxplots of the inter-amino acid distance distributions calculated with the Canberra and Clark distance functions.

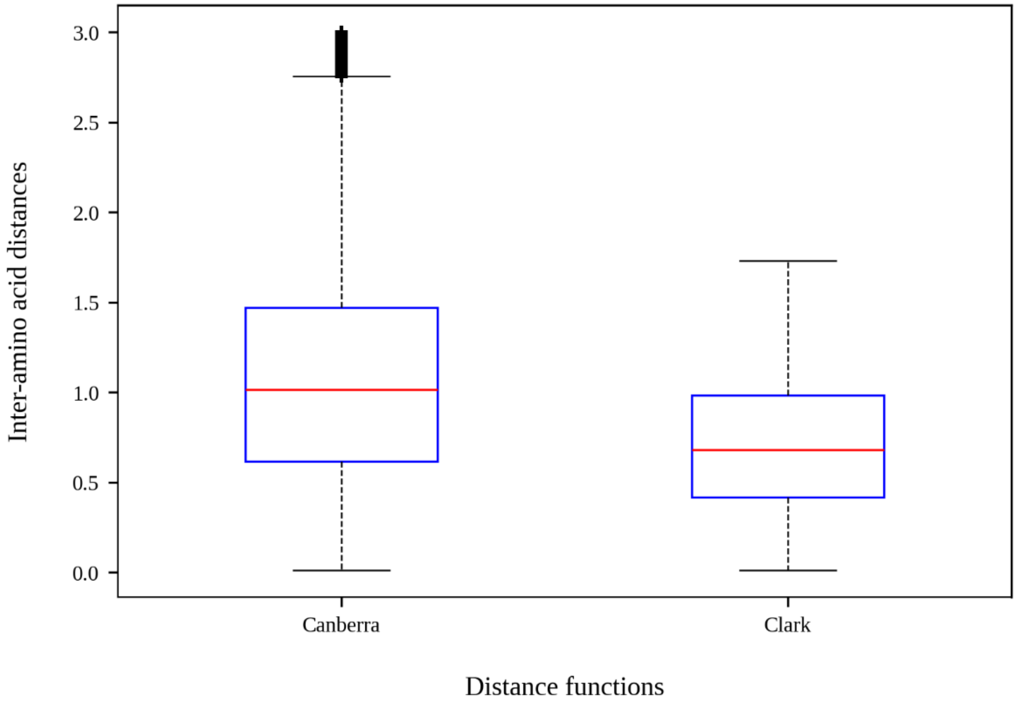

Figure S9. Boxplots of the density values corresponding to the graphs built with each of the distance thresholds studied in this work.

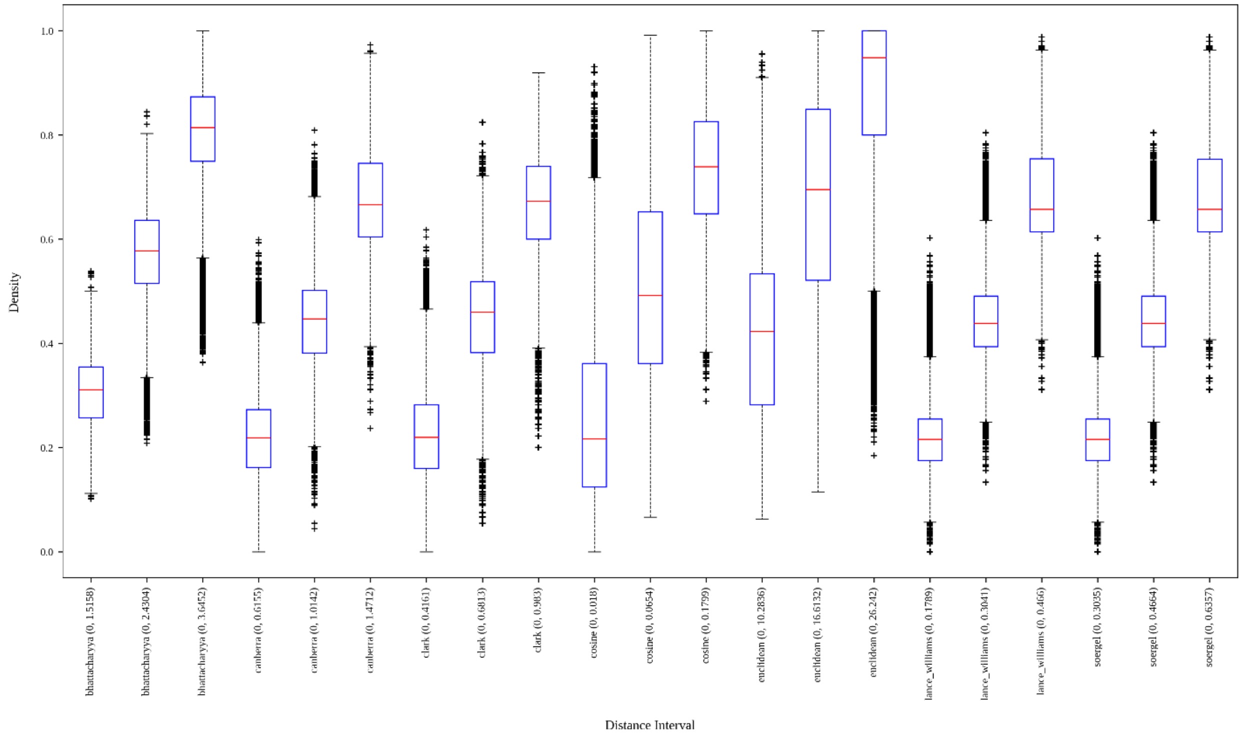

Figure S10. Boxplots of the perplexity values calculated for the peptide sequences extracted from the starPep database.

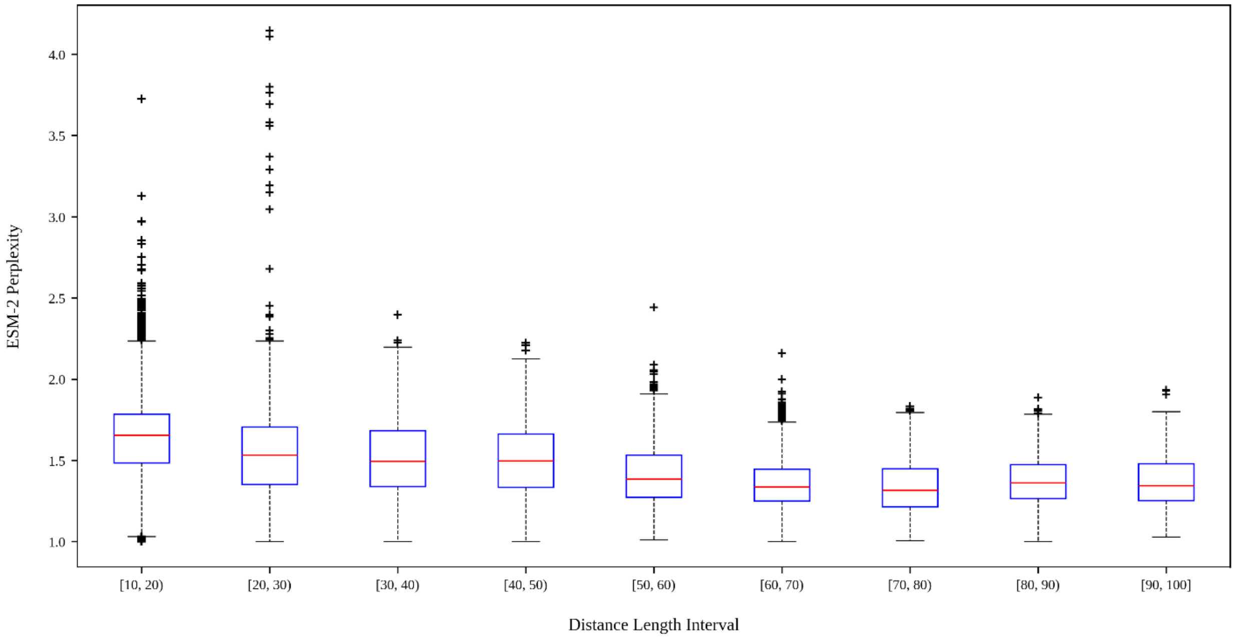

Figure S11. Boxplots of the perplexity values calculated for the sequences belonging to the AVPDiscover training set.

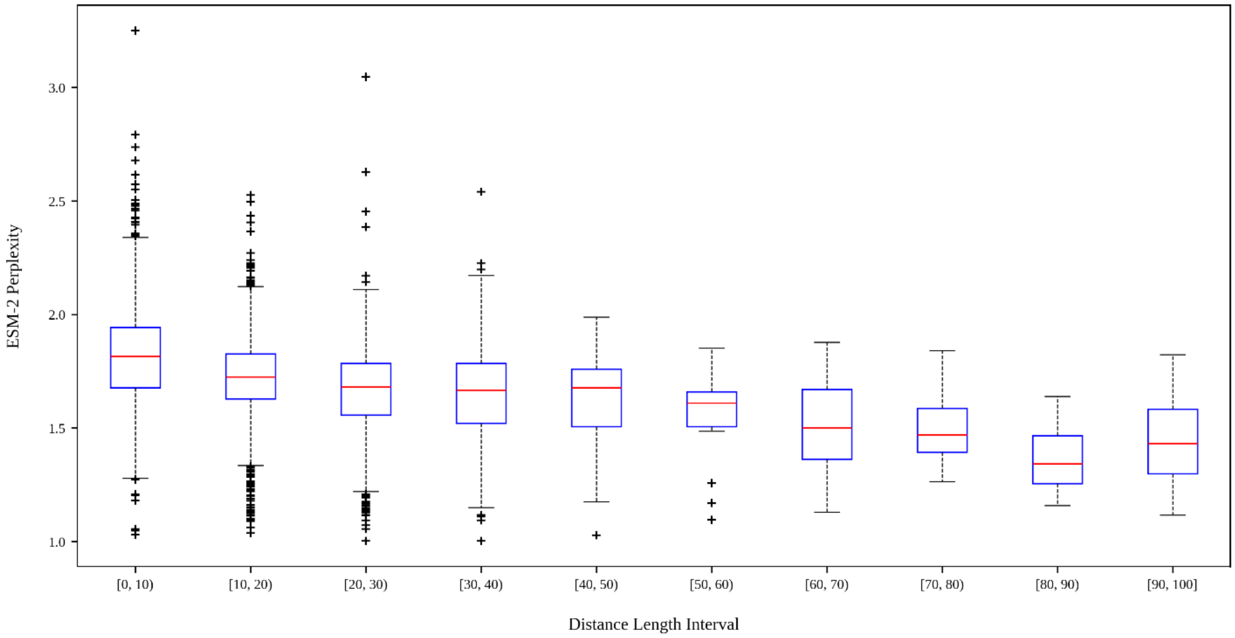

Figure S12. Boxplots of the perplexity values calculated for the sequences belonging to the AVPDiscover validation set.

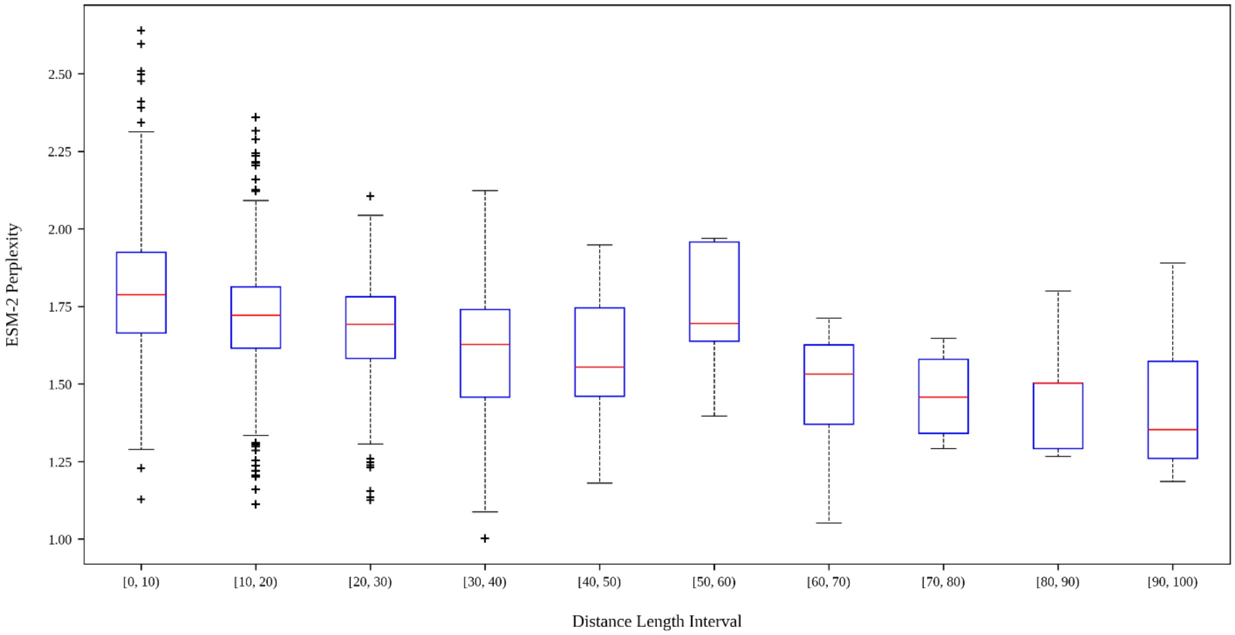

Figure S13. Boxplots of the perplexity values calculated for the sequences belonging to the AVPDiscover test set.

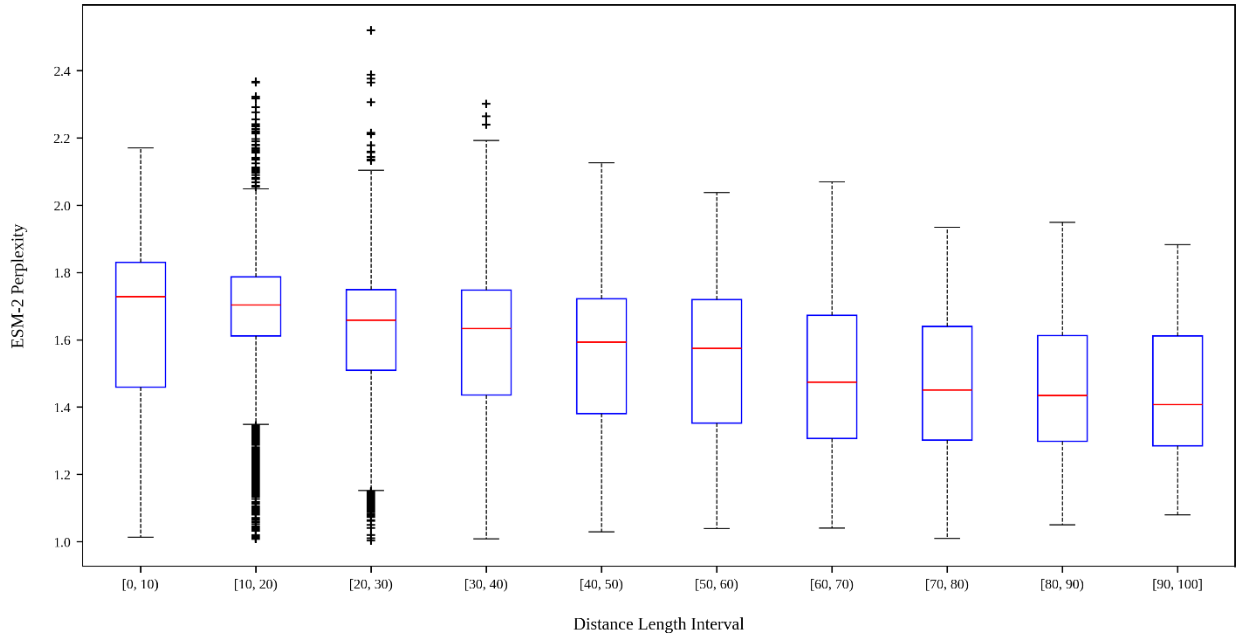

Files S1. FASTA files of the datasets used in this work:
https://drive.google.com/file/d/13ZIH-6I-bpLEjltB0WTW0Kq5fME8ghdW/view?usp=sharing

Data S1. Raw data of the cosine similarity coefficients calculated between each of the 30 graphs randomly built for each peptide sequence with regard to the graph derived from each predicted structure when applying the 21 distance-based thresholds considered:
https://drive.google.com/file/d/1U1L0Qeb9W3h6TBzOlb-Y6LLNX_hdEO67/view?usp=sharing

Data S2. Best model developed with the graphs derived from each of the following distance/threshold pairs: Cosine/0.018, Bhattacharyya/1.5158, Canberra/0.6155, Clark/0.4161, Euclidean/26.242, Lance-Williams/0.1789, and Soergel/0.3035.
https://drive.google.com/drive/folders/15OHABfl8I1uOI3WLkwByTZ-M2SWPXfZV?usp=sharing

Data S3. Predictions on the AVPDiscover test set and AVPDiscover reduced test set performed by the best model built with the graphs derived from each of the following distance/threshold pairs: Cosine/0.018, Bhattacharyya/1.5158, Canberra/0.6155, Clark/0.4161, Euclidean/26.242, Lance-Williams/0.1789, and Soergel/0.3035.
https://drive.google.com/drive/folders/1cYn5Asy4MTY-2b74LIW1YjRnzBEfLQRY?usp=sharing

Data S4. Predictions on the external set performed by the best model built with the graphs derived from each of the following distance/threshold pairs: Cosine/0.018, Bhattacharyya/1.5158, Canberra/0.6155, Clark/0.4161, Euclidean/26.242, Lance-Williams/0.1789, and Soergel/0.3035.

Also, it contains the results of the StackAVP and AI4AVP models from the literature**,** as well as the predictions of the combined models that were built by fusing the output of the individual models fed with the graphs built with the Euclidean/26.242 and Clark/0.4161 distance thresholds, from the Euclidean/26.242 and Cosine/0.018 distance thresholds, and from the Euclidean/26.242, Cosine/0.018, and Bhattacharyya /1.5158 distance thresholds, respectively.
https://drive.google.com/drive/folders/1BZhq58vyStpAJxL7lD5adejmwq8krCcJ?usp=drive_link

Section S1. Command line used to train the models. This command line was repeated 100-times for each distance/threshold pair.

```
dataset="/path/to/AVPDiscover(Training-Validation-Test).csv"
pdb_path="/path/to/the/output/directory/where/predicted/pdb/are/saved"
gdl_model_path="/path/to/the/directory/where/the/trained/models/are/saved"

tertiary_structure_method='esmfold'
esm2_representation="esm2_t33"
edge_construction_functions="distance_based_threshold"
distance_function="here must be specified the distance functions (e.g., euclidean)"
distance_threshold="here must be specified the distance threshold (e.g., 10)"
amino_acid_representation="CA"
number_of_heads=8
hidden_layer_dimension=128
learning_rate=0.0001
dropout_rate=0.25
batch_size=512
number_of_epoch=200

python train.py \
  --dataset "$dataset" \
  --pdb_path "$pdb_path" \
  --gdl_model_path="$gdl_model_path"  \
  --esm2_representation "$esm2_representation" \
  --edge_construction_functions="$edge_construction_functions" \
  --distance_function="$distance_function" \
  --distance_threshold="$distance_threshold" \
  --amino_acid_representation="$amino_acid_representation" \
  --number_of_heads="$number_of_heads" \
  --hidden_layer_dimension="$hidden_layer_dimension" \
  --learning_rate="$learning_rate" \
  --dropout_rate="$dropout_rate" \
  --batch_size="$batch_size" \
  --number_of_epoch="$number_of_epoch" \
  --add_self_loops \
  --use_edge_attr \
  --save_ckpt_per_epoch
```

Section S2. Command line used to run the test model with a given model.

```
dataset="/path/to/input/file.csv"
pdb_path="/path/to/the/output/directory/where/predicted/pdb/are/saved"
output_path ="/path/to/the/output/directory"
gdl_model_path="/path/to/a/given/model.pt"

tertiary_structure_method='esmfold'
batch_size=512

python test.py \
   --dataset="$dataset" \
   --pdb_path="$pdb_path" \
   --gdl_model_path="$gdl_model_path" \
   --tertiary_structure_method="$tertiary_structure_method"  \
   --output_path="$output_path"
```

When using a specific seed value, it should be used the next command line:

```
dataset="/path/to/input/file.csv"
pdb_path="/path/to/the/output/directory/where/predicted/pdb/are/saved"
output_path ="/path/to/the/output/directory"
gdl_model_path="/path/to/a/given/model.pt"

tertiary_structure_method='esmfold'
batch_size=512
seed=0

python test.py \
   --dataset="$dataset" \
   --pdb_path="$pdb_path" \
   --gdl_model_path="$gdl_model_path" \
   --tertiary_structure_method="$tertiary_structure_method"  \
   --output_path="$output_path"  \
   --seed="$seed"
```

Table S1. Measures of dispersion of the 21 similarity distributions obtained by calculating the cosine similarity between each of the 30 graphs randomly built for each peptide sequence with regard to the graph derived from each predicted structure when applying the 21 distance-based thresholds considered.

distance function	threshold	count	min	mean	std	p25	p50	p75	max	skewness	kurtosis
cosine	0.0180	1038150	-0.9648	0.4611	0.2773	0.3412	0.5109	0.6504	0.9947	-1.4341	5.9588
soergel	0.3035	1038540	-0.9702	0.4891	0.2506	0.3997	0.5175	0.6340	0.9956	-2.1083	10.4540
lance_williams	0.1789	1038540	-0.9608	0.4892	0.2507	0.3996	0.5177	0.6344	0.9971	-2.1078	10.4331
euclidean	10.2836	1039080	-0.8071	0.5043	0.1876	0.3661	0.4865	0.6391	0.9971	0.1889	2.4213
canberra	0.6155	1037880	-0.9752	0.5075	0.2475	0.4268	0.5400	0.6482	0.9977	-2.2149	10.7622
clark	0.4161	1038300	-0.9650	0.5128	0.2444	0.4343	0.5462	0.6527	0.9943	-2.1647	10.4266
euclidean	26.242	1039080	-0.6514	0.5165	0.3583	0.4836	0.6710	0.7348	0.9527	-1.6914	4.6346
bhattacharyya	1.5158	1039080	-0.9095	0.5165	0.1814	0.4011	0.5103	0.6354	0.9964	-0.3368	4.3199
cosine	0.0654	1039080	-0.9325	0.5850	0.2166	0.5086	0.6338	0.7186	0.9976	-2.1855	10.0128
euclidean	16.6132	1039080	-0.6347	0.5991	0.2025	0.5122	0.6261	0.7310	0.9848	-2.3827	12.6949
soergel	0.4664	1039080	-0.8708	0.6115	0.1426	0.5226	0.6150	0.7068	0.9972	-0.6220	5.7689
lance_williams	0.3041	1039080	-0.9821	0.6116	0.1424	0.5229	0.6151	0.7070	0.9971	-0.6256	5.7730
bhattacharyya	2.4304	1039080	-0.5901	0.6235	0.1321	0.5307	0.6189	0.7173	0.9967	0.0015	2.8390
canberra	1.0142	1039080	-0.9270	0.6303	0.1336	0.5519	0.6344	0.7167	0.9960	-1.2050	11.2226
clark	0.6813	1039080	-0.9559	0.6366	0.1419	0.5655	0.6445	0.7231	0.9940	-2.1676	17.0789
cosine	0.1799	1039080	-0.8849	0.6478	0.1944	0.6145	0.6914	0.7429	0.9937	-3.4155	17.8919
clark	0.9830	1039080	-0.7402	0.6706	0.1797	0.6443	0.6973	0.7464	0.9874	-4.5079	27.6936
lance_williams	0.4660	1039080	-0.8031	0.6733	0.1049	0.6057	0.6753	0.7433	0.9970	-0.3795	5.1690
soergel	0.6357	1039080	-0.5899	0.6734	0.1048	0.6060	0.6754	0.7433	0.9974	-0.3806	5.1390
canberra	1.4712	1039080	-0.7703	0.6785	0.1017	0.6189	0.6814	0.7427	0.9988	-1.1586	13.4108
bhattacharyya	3.6452	1039080	-0.6090	0.6818	0.1124	0.6218	0.6867	0.7502	0.9789	-2.9601	30.2385

Table S2. Measures of dispersion of the similarity distributions obtained by calculating the cosine similarity coefficient between the graph derived with a specific distance and threshold regarding the graph derived with another distance and threshold for each peptide sequence of the StarPep-derived dataset.

Distance function 1	Threshold 1	Distance function 2	Threshold 2	min	mean	std	p25	p50	p75	max
euclidean	26.242	cosine	0.018	-0.7454	0.5697	0.3850	0.3971	0.7092	0.8580	0.9918
euclidean	26.242	soergel	0.3035	-0.7454	0.6171	0.3248	0.5712	0.7418	0.8117	0.9808
euclidean	26.242	lance_williams	0.1789	-0.7454	0.6171	0.3248	0.5712	0.7418	0.8117	0.9831
euclidean	26.242	canberra	0.6155	-0.8264	0.6190	0.3584	0.5741	0.7663	0.8368	0.9814
cosine	0.018	cosine	0.1799	-0.8895	0.6225	0.3354	0.5439	0.7258	0.8570	0.9976
canberra	0.6155	cosine	0.018	-1.0000	0.6255	0.3517	0.5553	0.7571	0.8504	1.0000
euclidean	10.2836	euclidean	26.242	-0.2842	0.6272	0.1771	0.5250	0.6707	0.7625	0.9668
euclidean	26.242	clark	0.4161	-0.8013	0.6284	0.3522	0.5244	0.7753	0.8482	0.9809
clark	0.983	cosine	0.018	-0.8641	0.6367	0.3398	0.5432	0.7371	0.8749	1.0000
clark	0.4161	cosine	0.018	-1.0000	0.6391	0.3519	0.5693	0.7765	0.8622	1.0000
euclidean	16.6132	cosine	0.018	-0.8019	0.6432	0.3412	0.5933	0.7682	0.8539	0.9838
canberra	1.4712	cosine	0.018	-0.8940	0.6471	0.3166	0.5618	0.7405	0.8642	1.0000
euclidean	10.2836	cosine	0.018	-0.8847	0.6490	0.2938	0.6025	0.7519	0.8303	0.9786
soergel	0.6357	cosine	0.018	-0.8735	0.6494	0.3178	0.5640	0.7505	0.8654	0.9994
lance_williams	0.466	cosine	0.018	-0.8735	0.6494	0.3179	0.5641	0.7505	0.8654	0.9994
euclidean	26.242	bhattacharyya	1.5158	-0.5355	0.6506	0.2150	0.5897	0.7326	0.7977	0.9520
bhattacharyya	3.6452	cosine	0.018	-0.8352	0.6526	0.3226	0.5722	0.7581	0.8677	0.9946
lance_williams	0.1789	cosine	0.018	-1.0000	0.6532	0.3361	0.5978	0.7847	0.8607	1.0000
soergel	0.3035	cosine	0.018	-1.0000	0.6532	0.3360	0.5977	0.7847	0.8607	1.0000
canberra	1.0142	cosine	0.018	-0.9272	0.6558	0.3212	0.5892	0.7594	0.8673	0.9995
clark	0.6813	cosine	0.018	-0.9069	0.6594	0.3256	0.5920	0.7687	0.8747	1.0000
cosine	0.018	cosine	0.0654	-0.9430	0.6608	0.3344	0.6100	0.7839	0.8817	0.9993
lance_williams	0.3041	cosine	0.018	-0.8969	0.6660	0.3162	0.6077	0.7735	0.8775	0.9991
soergel	0.4664	cosine	0.018	-0.8969	0.6661	0.3161	0.6078	0.7736	0.8775	0.9991
bhattacharyya	2.4304	cosine	0.018	-0.8677	0.6673	0.3136	0.6066	0.7783	0.8731	0.9957
bhattacharyya	1.5158	cosine	0.018	-0.9233	0.6688	0.3019	0.6290	0.7878	0.8516	1.0000
lance_williams	0.1789	cosine	0.1799	-0.9026	0.6861	0.2844	0.6930	0.7611	0.8187	0.9714

soergel	0.3035	cosine	0.1799	-0.9026	0.6862	0.2844	0.6929	0.7611	0.8187	0.9714
lance_williams	0.1789	clark	0.983	-0.8985	0.6946	0.2875	0.6785	0.7703	0.8339	0.9790
clark	0.983	soergel	0.3035	-0.8985	0.6946	0.2875	0.6785	0.7703	0.8339	0.9790
canberra	0.6155	cosine	0.1799	-0.8893	0.6992	0.2864	0.7139	0.7847	0.8380	0.9783
euclidean	26.242	cosine	0.0654	-0.6413	0.6997	0.3128	0.4803	0.8410	0.9278	0.9991
**euclidean**	**10.2836**	**canberra**	**0.6155**	**-0.9154**	**0.7057**	**0.2633**	**0.6850**	**0.7682**	**0.8389**	**0.9857**
clark	0.4161	cosine	0.1799	-0.9009	0.7070	0.2831	0.7218	0.7955	0.8483	0.9807
canberra	1.4712	soergel	0.3035	-0.8928	0.7079	0.2686	0.6899	0.7700	0.8325	0.9757
canberra	1.4712	lance_williams	0.1789	-0.8928	0.7079	0.2686	0.6899	0.7701	0.8325	0.9757
canberra	0.6155	clark	0.983	-0.8891	0.7088	0.2961	0.7101	0.7918	0.8509	0.9890
canberra	0.6155	cosine	0.0654	-0.9621	0.7098	0.2923	0.7195	0.8070	0.8616	0.9947
soergel	0.3035	bhattacharyya	3.6452	-0.8340	0.7127	0.2788	0.7001	0.7795	0.8394	0.9868
lance_williams	0.1789	bhattacharyya	3.6452	-0.8340	0.7127	0.2788	0.7001	0.7795	0.8394	0.9868
euclidean	10.2836	clark	0.4161	-0.9006	0.7161	0.2570	0.6953	0.7828	0.8500	0.9875
lance_williams	0.466	soergel	0.3035	-0.8999	0.7165	0.2697	0.7007	0.7786	0.8416	0.9752
lance_williams	0.1789	lance_williams	0.466	-0.8999	0.7165	0.2697	0.7007	0.7786	0.8417	0.9752
soergel	0.3035	soergel	0.6357	-0.8999	0.7165	0.2697	0.7008	0.7786	0.8417	0.9752
lance_williams	0.1789	soergel	0.6357	-0.8999	0.7165	0.2697	0.7007	0.7786	0.8417	0.9752
lance_williams	0.1789	cosine	0.0654	-0.9866	0.7168	0.2861	0.7317	0.8011	0.8541	1.0000
soergel	0.3035	cosine	0.0654	-0.9866	0.7168	0.2861	0.7317	0.8011	0.8541	1.0000
clark	0.4161	clark	0.983	-0.8657	0.7172	0.2909	0.7168	0.8010	0.8637	0.9842
euclidean	10.2836	clark	0.983	-0.4909	0.7178	0.1587	0.6189	0.7452	0.8389	0.9874
canberra	0.6155	bhattacharyya	1.5158	-0.9541	0.7188	0.2718	0.7131	0.8006	0.8610	0.9974
clark	0.4161	cosine	0.0654	-0.8899	0.7198	0.2910	0.7343	0.8214	0.8725	0.9869
canberra	0.6155	soergel	0.3035	-1.0000	0.7200	0.3047	0.7025	0.8134	0.8853	1.0000
canberra	0.6155	lance_williams	0.1789	-1.0000	0.7200	0.3047	0.7026	0.8135	0.8853	1.0000
clark	0.4161	soergel	0.3035	-1.0000	0.7219	0.3097	0.7185	0.8234	0.8849	1.0000
lance_williams	0.1789	clark	0.4161	-1.0000	0.7219	0.3097	0.7186	0.8234	0.8850	1.0000
canberra	0.6155	canberra	1.4712	-0.9133	0.7228	0.2688	0.7139	0.7919	0.8509	0.9828
euclidean	10.2836	cosine	0.1799	-0.5305	0.7261	0.1632	0.6230	0.7435	0.8524	1.0000
canberra	0.6155	lance_williams	0.466	-0.8951	0.7291	0.2707	0.7253	0.7992	0.8567	0.9874

canberra	0.6155	soergel	0.6357	-0.8951	0.7291	0.2707	0.7252	0.7992	0.8568	0.9874
canberra	0.6155	bhattacharyya	3.6452	-0.8404	0.7301	0.2781	0.7283	0.8004	0.8570	0.9885
canberra	1.4712	clark	0.4161	-0.9071	0.7301	0.2663	0.7216	0.8005	0.8610	0.9832
clark	0.4161	bhattacharyya	1.5158	-0.9221	0.7310	0.2651	0.7355	0.8164	0.8678	0.9918
euclidean	16.6132	canberra	0.6155	-0.8584	0.7314	0.3009	0.7416	0.8190	0.8718	0.9870
euclidean	16.6132	soergel	0.3035	-0.8415	0.7316	0.2824	0.7216	0.8106	0.8638	0.9862
euclidean	16.6132	lance_williams	0.1789	-0.8415	0.7316	0.2824	0.7216	0.8106	0.8638	0.9862
canberra	1.0142	soergel	0.3035	-0.9551	0.7323	0.2788	0.7202	0.7977	0.8647	1.0000
canberra	1.0142	lance_williams	0.1789	-0.9551	0.7324	0.2788	0.7203	0.7977	0.8647	1.0000
clark	0.6813	soergel	0.3035	-0.9732	0.7330	0.2787	0.7268	0.8011	0.8619	1.0000
lance_williams	0.1789	clark	0.6813	-0.9732	0.7330	0.2787	0.7268	0.8012	0.8620	1.0000
euclidean	16.6132	clark	0.4161	-0.8564	0.7342	0.2987	0.7446	0.8225	0.8771	0.9913
euclidean	26.242	cosine	0.1799	-0.3926	0.7349	0.3334	0.4911	0.9074	0.9547	1.0000
clark	0.983	bhattacharyya	1.5158	-0.8627	0.7360	0.1651	0.6910	0.7671	0.8320	0.9859
lance_williams	0.466	clark	0.4161	-0.8999	0.7361	0.2689	0.7340	0.8085	0.8663	0.9889
clark	0.4161	soergel	0.6357	-0.8999	0.7361	0.2688	0.7340	0.8084	0.8664	0.9889
euclidean	26.242	canberra	1.0142	-0.6694	0.7371	0.3069	0.7920	0.8845	0.9182	0.9964
clark	0.4161	bhattacharyya	3.6452	-0.8210	0.7371	0.2758	0.7367	0.8103	0.8674	0.9916
euclidean	10.2836	lance_williams	0.1789	-0.8913	0.7382	0.2763	0.7285	0.8045	0.8669	0.9867
euclidean	10.2836	soergel	0.3035	-0.8913	0.7382	0.2763	0.7285	0.8045	0.8669	0.9867
euclidean	10.2836	cosine	0.0654	-0.7363	0.7410	0.1692	0.6645	0.7796	0.8556	0.9927
bhattacharyya	1.5158	cosine	0.1799	-0.8593	0.7419	0.1600	0.7011	0.7699	0.8299	0.9957
euclidean	26.242	soergel	0.4664	-0.5418	0.7425	0.2593	0.7668	0.8672	0.9000	0.9970
euclidean	26.242	lance_williams	0.3041	-0.5418	0.7425	0.2592	0.7668	0.8672	0.9000	0.9970
euclidean	26.242	clark	0.6813	-0.6086	0.7428	0.3132	0.7998	0.8957	0.9302	0.9955
canberra	0.6155	canberra	1.0142	-0.9551	0.7434	0.2772	0.7400	0.8179	0.8785	1.0000
euclidean	10.2836	canberra	1.4712	-0.5909	0.7436	0.1318	0.6422	0.7611	0.8513	0.9975
canberra	0.6155	clark	0.6813	-0.9426	0.7444	0.2787	0.7502	0.8221	0.8751	0.9897
euclidean	26.242	bhattacharyya	2.4304	-0.3624	0.7467	0.2686	0.7959	0.8790	0.9092	0.9971
lance_williams	0.1789	bhattacharyya	1.5158	-0.9494	0.7475	0.2827	0.7563	0.8280	0.8820	0.9947
soergel	0.3035	bhattacharyya	1.5158	-0.9494	0.7475	0.2827	0.7563	0.8280	0.8820	0.9947

soergel	0.3035	soergel	0.4664	-0.9616	0.7478	0.2809	0.7436	0.8200	0.8828	0.9909
lance_williams	0.3041	soergel	0.3035	-0.9616	0.7478	0.2809	0.7436	0.8200	0.8828	0.9909
lance_williams	0.1789	soergel	0.4664	-0.9616	0.7478	0.2809	0.7438	0.8200	0.8828	0.9909
lance_williams	0.1789	lance_williams	0.3041	-0.9616	0.7478	0.2809	0.7436	0.8200	0.8828	0.9909
canberra	0.6155	lance_williams	0.3041	-0.9387	0.7492	0.2736	0.7508	0.8269	0.8864	0.9926
canberra	1.0142	clark	0.4161	-0.9551	0.7492	0.2740	0.7486	0.8237	0.8834	1.0000
canberra	0.6155	soergel	0.4664	-0.9387	0.7492	0.2735	0.7508	0.8270	0.8865	0.9926
euclidean	16.6132	euclidean	26.242	-0.2318	0.7504	0.3056	0.7801	0.8777	0.9349	1.0000
soergel	0.3035	bhattacharyya	2.4304	-0.8830	0.7508	0.2760	0.7460	0.8208	0.8784	0.9932
lance_williams	0.1789	bhattacharyya	2.4304	-0.8830	0.7508	0.2760	0.7460	0.8208	0.8784	0.9932
euclidean	26.242	canberra	1.4712	-0.4980	0.7517	0.3476	0.8218	0.9294	0.9569	1.0000
clark	0.4161	clark	0.6813	-0.9426	0.7531	0.2744	0.7609	0.8317	0.8836	0.9916
canberra	0.6155	bhattacharyya	2.4304	-0.8925	0.7548	0.2714	0.7571	0.8299	0.8853	0.9951
lance_williams	0.3041	clark	0.4161	-0.9630	0.7564	0.2683	0.7613	0.8334	0.8909	0.9977
clark	0.4161	soergel	0.4664	-0.9630	0.7564	0.2683	0.7614	0.8333	0.8909	0.9977
euclidean	10.2836	bhattacharyya	3.6452	-0.3641	0.7576	0.1402	0.6561	0.7751	0.8707	1.0000
canberra	1.4712	bhattacharyya	1.5158	-0.6289	0.7606	0.1379	0.7068	0.7774	0.8412	0.9897
euclidean	26.242	clark	0.983	-0.4465	0.7610	0.3636	0.8497	0.9458	0.9701	0.9989
clark	0.4161	bhattacharyya	2.4304	-0.8925	0.7612	0.2670	0.7676	0.8361	0.8897	0.9930
euclidean	10.2836	soergel	0.6357	-0.1301	0.7632	0.1327	0.6610	0.7852	0.8748	1.0000
euclidean	10.2836	lance_williams	0.466	-0.1301	0.7632	0.1327	0.6611	0.7852	0.8748	1.0000
bhattacharyya	1.5158	bhattacharyya	3.6452	-0.5544	0.7646	0.1425	0.7198	0.7822	0.8435	0.9744
euclidean	10.2836	clark	0.6813	-0.8900	0.7653	0.1371	0.6809	0.7868	0.8686	0.9849
euclidean	10.2836	canberra	1.0142	-0.8900	0.7683	0.1267	0.6859	0.7842	0.8653	0.9855
bhattacharyya	1.5158	cosine	0.0654	-0.9036	0.7688	0.1720	0.7438	0.8063	0.8599	0.9992
euclidean	26.242	soergel	0.6357	-0.4892	0.7693	0.3106	0.8353	0.9280	0.9536	0.9997
euclidean	26.242	lance_williams	0.466	-0.4892	0.7693	0.3106	0.8353	0.9280	0.9536	0.9997
euclidean	26.242	bhattacharyya	3.6452	-0.3116	0.7695	0.3508	0.8879	0.9544	0.9715	1.0000
soergel	0.6357	bhattacharyya	1.5158	-0.6188	0.7737	0.1371	0.7242	0.7908	0.8518	0.9878
lance_williams	0.466	bhattacharyya	1.5158	-0.6188	0.7737	0.1371	0.7241	0.7908	0.8518	0.9878
canberra	0.6155	clark	0.4161	-1.0000	0.7823	0.2721	0.7685	0.8569	0.9217	1.0000

clark	0.6813	bhattacharyya	1.5158	-0.7119	0.7834	0.1512	0.7430	0.8062	0.8633	1.0000
euclidean	16.6132	bhattacharyya	1.5158	-0.5355	0.7840	0.1649	0.7560	0.8187	0.8708	0.9907
canberra	1.0142	bhattacharyya	1.5158	-0.8866	0.7840	0.1487	0.7375	0.8045	0.8641	1.0000
euclidean	16.6132	cosine	0.0654	-0.6413	0.7843	0.2134	0.7671	0.8484	0.8988	0.9958
euclidean	10.2836	euclidean	16.6132	-0.2842	0.7844	0.1339	0.7416	0.8018	0.8642	0.9731
clark	0.983	cosine	0.0654	-0.6941	0.7990	0.2458	0.7628	0.8807	0.9466	1.0000
euclidean	10.2836	soergel	0.4664	-0.6753	0.8000	0.1199	0.7240	0.8227	0.8920	1.0000
**euclidean**	**10.2836**	**lance_williams**	**0.3041**	**-0.6753**	**0.8000**	**0.1199**	**0.7240**	**0.8227**	**0.8921**	**1.0000**
cosine	0.0654	cosine	0.1799	-0.7866	0.8100	0.2628	0.8032	0.8902	0.9531	1.0000
canberra	1.0142	cosine	0.0654	-0.9307	0.8119	0.2033	0.8004	0.8677	0.9127	1.0000
lance_williams	0.3041	bhattacharyya	1.5158	-0.9074	0.8144	0.1326	0.7692	0.8344	0.8878	1.0000
soergel	0.4664	bhattacharyya	1.5158	-0.9074	0.8144	0.1326	0.7693	0.8344	0.8878	1.0000
euclidean	16.6132	cosine	0.1799	-0.5298	0.8167	0.1957	0.7894	0.8760	0.9286	0.9998
canberra	1.4712	cosine	0.0654	-0.7911	0.8180	0.2062	0.7833	0.8785	0.9334	1.0000
clark	0.6813	cosine	0.0654	-0.9141	0.8182	0.2126	0.8111	0.8800	0.9234	1.0000
bhattacharyya	1.5158	bhattacharyya	2.4304	-0.6629	0.8185	0.1227	0.7817	0.8320	0.8812	0.9915
euclidean	10.2836	bhattacharyya	2.4304	-0.4123	0.8201	0.1017	0.7411	0.8332	0.9037	0.9994
bhattacharyya	3.6452	cosine	0.0654	-0.6910	0.8217	0.2114	0.7949	0.8893	0.9336	0.9993
soergel	0.6357	cosine	0.0654	-0.7358	0.8251	0.1990	0.7955	0.8855	0.9318	1.0000
lance_williams	0.466	cosine	0.0654	-0.7358	0.8251	0.1990	0.7956	0.8855	0.9318	1.0000
bhattacharyya	2.4304	cosine	0.0654	-0.8039	0.8252	0.1812	0.8190	0.8710	0.9091	0.9974
lance_williams	0.3041	cosine	0.0654	-0.8232	0.8278	0.1837	0.8243	0.8739	0.9141	0.9988
soergel	0.4664	cosine	0.0654	-0.8232	0.8279	0.1837	0.8244	0.8739	0.9142	0.9988
euclidean	16.6132	clark	0.983	-0.4719	0.8339	0.2109	0.8105	0.9038	0.9441	0.9972
euclidean	10.2836	bhattacharyya	1.5158	-0.7423	0.8436	0.1118	0.8139	0.8573	0.8996	0.9942
lance_williams	0.3041	cosine	0.1799	-0.5352	0.8465	0.1612	0.8412	0.8809	0.9146	1.0000
soergel	0.4664	cosine	0.1799	-0.5352	0.8465	0.1612	0.8413	0.8809	0.9146	1.0000
euclidean	16.6132	canberra	1.0142	-0.8292	0.8472	0.1709	0.8346	0.8866	0.9240	0.9937
euclidean	16.6132	clark	0.6813	-0.8292	0.8490	0.1765	0.8381	0.8925	0.9296	0.9928
canberra	1.0142	cosine	0.1799	-0.9028	0.8496	0.1747	0.8484	0.8923	0.9243	0.9950
euclidean	16.6132	canberra	1.4712	-0.4980	0.8503	0.1754	0.8241	0.9016	0.9399	1.0000

euclidean	16.6132	soergel	0.4664	-0.5665	0.8508	0.1465	0.8379	0.8838	0.9154	0.9995
euclidean	16.6132	lance_williams	0.3041	-0.5665	0.8508	0.1465	0.8379	0.8838	0.9154	0.9995
lance_williams	0.3041	clark	0.983	-0.7726	0.8509	0.1487	0.8410	0.8845	0.9169	1.0000
clark	0.983	soergel	0.4664	-0.7726	0.8510	0.1487	0.8410	0.8845	0.9169	1.0000
bhattacharyya	2.4304	cosine	0.1799	-0.5653	0.8528	0.1533	0.8405	0.8872	0.9215	0.9998
clark	0.6813	cosine	0.1799	-0.8629	0.8561	0.1896	0.8623	0.9053	0.9349	0.9968
clark	0.983	bhattacharyya	2.4304	-0.5175	0.8582	0.1421	0.8473	0.8896	0.9230	1.0000
clark	0.983	cosine	0.1799	-0.6307	0.8658	0.2352	0.8928	0.9370	0.9649	1.0000
euclidean	16.6132	lance_williams	0.466	-0.4892	0.8660	0.1573	0.8423	0.9155	0.9475	1.0000
euclidean	16.6132	soergel	0.6357	-0.4892	0.8660	0.1572	0.8424	0.9155	0.9475	1.0000
canberra	1.0142	clark	0.983	-0.8897	0.8689	0.1623	0.8665	0.9060	0.9362	1.0000
euclidean	16.6132	bhattacharyya	3.6452	-0.3402	0.8719	0.1862	0.8452	0.9320	0.9622	1.0000
canberra	1.4712	lance_williams	0.3041	-0.5734	0.8723	0.0909	0.8460	0.8866	0.9201	0.9977
canberra	1.4712	soergel	0.4664	-0.5734	0.8723	0.0909	0.8460	0.8867	0.9201	0.9977
clark	0.6813	clark	0.983	-0.7695	0.8758	0.1775	0.8788	0.9191	0.9481	1.0000
lance_williams	0.3041	clark	0.6813	-0.9175	0.8799	0.1244	0.8618	0.9012	0.9373	1.0000
clark	0.6813	soergel	0.4664	-0.9175	0.8799	0.1242	0.8618	0.9012	0.9373	1.0000
lance_williams	0.3041	bhattacharyya	3.6452	-0.5327	0.8803	0.1026	0.8603	0.8940	0.9233	0.9991
soergel	0.4664	bhattacharyya	3.6452	-0.5327	0.8803	0.1026	0.8604	0.8941	0.9233	0.9991
canberra	1.0142	soergel	0.4664	-0.8745	0.8816	0.1147	0.8551	0.9000	0.9428	1.0000
canberra	1.0142	lance_williams	0.3041	-0.8745	0.8816	0.1147	0.8550	0.9000	0.9428	1.0000
canberra	1.4712	cosine	0.1799	-0.6118	0.8833	0.1782	0.8877	0.9274	0.9550	1.0000
canberra	1.0142	bhattacharyya	2.4304	-0.9041	0.8833	0.0888	0.8568	0.8941	0.9320	0.9979
canberra	1.4712	bhattacharyya	2.4304	-0.3580	0.8834	0.0691	0.8529	0.8922	0.9275	1.0000
clark	0.6813	bhattacharyya	2.4304	-0.9041	0.8847	0.1056	0.8653	0.9003	0.9340	0.9987
canberra	1.0142	canberra	1.4712	-0.9384	0.8851	0.0995	0.8611	0.9035	0.9361	0.9987
bhattacharyya	3.6452	cosine	0.1799	-0.4728	0.8868	0.1849	0.8954	0.9316	0.9568	1.0000
lance_williams	0.3041	lance_williams	0.466	-0.7491	0.8870	0.0818	0.8625	0.8944	0.9287	0.9979
lance_williams	0.3041	soergel	0.6357	-0.7491	0.8870	0.0818	0.8626	0.8944	0.9286	0.9979
lance_williams	0.466	soergel	0.4664	-0.7491	0.8871	0.0818	0.8626	0.8944	0.9286	0.9981
soergel	0.4664	soergel	0.6357	-0.7491	0.8871	0.0818	0.8627	0.8945	0.9286	0.9981

euclidean	16.6132	bhattacharyya	2.4304	-0.3895	0.8877	0.1349	0.8834	0.9139	0.9415	0.9990
canberra	1.4712	clark	0.6813	-0.9384	0.8908	0.1229	0.8729	0.9140	0.9458	0.9990
lance_williams	0.466	cosine	0.1799	-0.4838	0.8912	0.1688	0.8974	0.9304	0.9545	1.0000
soergel	0.6357	cosine	0.1799	-0.4838	0.8912	0.1688	0.8974	0.9305	0.9545	1.0000
canberra	1.0142	bhattacharyya	3.6452	-0.6828	0.8942	0.1094	0.8770	0.9098	0.9386	0.9974
canberra	1.0142	soergel	0.6357	-0.7857	0.8947	0.0916	0.8730	0.9086	0.9395	0.9985
canberra	1.0142	lance_williams	0.466	-0.7857	0.8947	0.0916	0.8730	0.9087	0.9396	0.9985
lance_williams	0.3041	bhattacharyya	2.4304	-0.8026	0.8967	0.0806	0.8680	0.9040	0.9416	0.9989
soergel	0.4664	bhattacharyya	2.4304	-0.8026	0.8967	0.0806	0.8680	0.9040	0.9416	0.9989
clark	0.6813	soergel	0.6357	-0.7857	0.8994	0.1138	0.8846	0.9182	0.9469	1.0000
lance_williams	0.466	clark	0.6813	-0.7857	0.8994	0.1139	0.8845	0.9182	0.9470	1.0000
**clark**	**0.6813**	**bhattacharyya**	**3.6452**	**-0.7587**	**0.9002**	**0.1313**	**0.8890**	**0.9219**	**0.9502**	**0.9988**
bhattacharyya	2.4304	bhattacharyya	3.6452	-0.4100	0.9003	0.0784	0.8766	0.9091	0.9387	0.9988
lance_williams	0.466	bhattacharyya	2.4304	-0.3947	0.9006	0.0588	0.8715	0.9058	0.9408	1.0000
soergel	0.6357	bhattacharyya	2.4304	-0.3947	0.9006	0.0588	0.8716	0.9059	0.9407	1.0000
canberra	1.0142	clark	0.6813	-0.8629	0.9039	0.1189	0.8851	0.9259	0.9597	1.0000
clark	0.983	soergel	0.6357	-0.5942	0.9109	0.1622	0.9208	0.9469	0.9668	1.0000
lance_williams	0.466	clark	0.983	-0.5942	0.9109	0.1622	0.9208	0.9470	0.9669	1.0000
canberra	1.4712	clark	0.983	-0.5635	0.9117	0.1743	0.9224	0.9525	0.9730	1.0000
clark	0.983	bhattacharyya	3.6452	-0.4759	0.9132	0.1791	0.9304	0.9539	0.9708	0.9990
canberra	1.4712	bhattacharyya	3.6452	-0.4639	0.9321	0.0897	0.9186	0.9457	0.9664	0.9997
canberra	1.4712	soergel	0.6357	-0.3530	0.9345	0.0649	0.9162	0.9469	0.9701	1.0000
canberra	1.4712	lance_williams	0.466	-0.3530	0.9346	0.0649	0.9163	0.9470	0.9702	1.0000
soergel	0.6357	bhattacharyya	3.6452	-0.4270	0.9395	0.0824	0.9270	0.9495	0.9690	1.0000
lance_williams	0.466	bhattacharyya	3.6452	-0.4270	0.9395	0.0825	0.9270	0.9496	0.9690	1.0000
lance_williams	0.3041	soergel	0.4664	0.6529	0.9986	0.0109	1.0000	1.0000	1.0000	1.0000
lance_williams	0.466	soergel	0.6357	0.7961	0.9991	0.0069	1.0000	1.0000	1.0000	1.0000
lance_williams	0.1789	soergel	0.3035	0.6360	0.9996	0.0075	1.0000	1.0000	1.0000	1.0000

Table S3. Mathews Correlation Coefficients obtained on the AVPDiscover validation set by the 100 models trained on the AVPDiscover training set by using graphs derived from the following distance/threshold pairs: Cosine/0.018, Bhattacharyya/1.5158, Canberra/0.6155, Clark/0.4161, Euclidean/26.242, Lance-Williams/0.1789, and Soergel/0.3035. The measures of dispersion are shown at the bottom of this table.

No. Model	Cosine 0.018	Bhattacharyya 1.5158	Canberra 0.6155	Clark 0.4161	Euclidean 26.242	Lance-Williams 0.1789	Soergel 0.3035
0	0.5942	0.6565	0.6100	0.6166	0.6727	0.6319	0.6117
1	0.6309	0.6309	0.6405	0.6264	0.6694	0.6477	0.6198
10	0.5823	0.6665	0.5683	0.6219	0.6731	0.5987	0.6190
11	0.6454	0.6152	0.5590	0.6277	0.6742	0.6373	0.5875
12	0.6150	0.6184	0.6170	0.6424	0.6825	0.6141	0.5843
13	0.6357	0.5912	0.5692	0.5913	0.6728	0.6389	0.6102
14	0.6164	0.6407	0.5714	0.5876	0.6663	0.6245	0.6116
15	0.6369	0.6717	0.6107	0.5715	0.6823	0.5495	0.5733
16	0.6340	0.6079	0.5891	0.6330	0.6746	0.5840	0.6327
17	0.6178	0.6309	0.6020	0.6158	0.6633	0.5655	0.6269
18	0.6180	0.6601	0.5964	0.6201	0.6839	0.6373	0.5826
19	0.6334	0.6244	0.6329	0.5896	0.6975	0.6395	0.6357
2	0.5769	0.6174	0.6036	0.6458	0.6966	0.6166	0.6212
20	0.6324	0.6324	0.6173	0.6194	0.6870	0.6357	0.6374
21	0.6324	0.6389	0.5772	0.6022	0.6665	0.6196	0.5731
22	0.6362	0.6202	0.5833	0.5686	0.6987	0.5811	0.6028
23	0.6533	0.6540	0.5572	0.6164	0.6669	0.6087	0.5956
24	0.6535	0.6266	0.6198	0.5546	0.6730	0.6152	0.6262
25	0.5752	0.6360	0.6240	0.6374	0.6666	0.6180	0.6038
26	0.6249	0.6566	0.6239	0.6393	0.6646	0.6006	0.6421
27	0.6434	0.6464	0.6328	0.6485	0.6912	0.6135	0.6229
28	0.6361	0.6501	0.6344	0.6565	0.6822	0.5784	0.5973
29	0.6196	0.6386	0.6329	0.6279	0.6664	0.6421	0.6213
3	0.6296	0.6552	0.5942	0.6229	0.6648	0.6517	0.6171
30	0.6263	0.6352	0.6165	0.6387	0.6941	0.6277	0.5956
31	0.6198	0.6329	0.6025	0.5992	0.6808	0.5914	0.6359

32	0.6277	0.6586	0.6037	0.6123	0.6694	0.5959	0.6475
33	0.6323	0.6679	0.6197	0.5676	0.6726	0.5847	0.6090
34	0.6247	0.6502	0.6205	0.5701	0.6597	0.6112	0.5867
35	0.6327	0.6790	0.5330	0.5779	0.6737	0.5898	0.6470
36	0.6133	0.6681	0.5903	0.6294	0.6577	0.5634	0.5845
37	0.6231	0.6308	0.6036	0.6135	0.6840	0.6340	0.6100
38	0.6180	0.6218	0.6437	0.6344	0.6565	0.6135	0.6230
39	0.6116	0.6326	0.5722	0.6228	0.6808	0.5764	0.6405
4	0.6330	0.6571	0.6228	0.6329	0.6758	0.6149	0.6488
40	0.6533	0.6743	0.6407	0.5895	0.6461	0.6382	0.5847
41	0.6178	0.6511	0.6485	0.6220	0.6873	0.6283	0.6229
42	0.6214	0.6645	0.5795	0.6174	0.6618	0.6405	0.6084
43	0.6311	0.6341	0.5676	0.6235	0.6661	0.6127	0.6487
44	0.6356	0.6326	0.6047	0.5797	0.6772	0.6043	0.6278
45	0.5734	0.6293	0.5404	0.5484	0.6731	0.6264	0.6324
46	0.6245	0.6742	0.6328	0.5649	0.6806	0.6201	0.6044
47	0.6099	0.6457	0.6424	0.5754	0.6726	0.6019	0.6230
48	0.5975	0.6731	0.5956	0.5512	0.6742	0.5877	0.6266
49	0.5782	0.6425	0.6184	0.6084	0.6793	0.6127	0.6036
5	0.5931	0.6711	0.6358	0.5370	0.7017	0.6168	0.6390
50	0.6215	0.6319	0.6164	0.6050	0.6731	0.5859	0.5973
51	0.6550	0.6364	0.6357	0.6108	0.6696	0.6191	0.6182
52	0.6019	0.6677	0.6535	0.6166	0.6613	0.5956	0.5877
53	0.5571	0.6357	0.5886	0.6127	0.6742	0.5977	0.5794
54	0.6245	0.6503	0.5988	0.6244	0.6822	0.6308	0.6137
55	0.6087	0.6904	0.6164	0.6116	0.6854	0.6559	0.5883
56	0.6201	0.6907	0.6313	0.5841	0.6746	0.6407	0.6422
57	0.5760	0.6421	0.6051	0.6552	0.6597	0.6337	0.6019
58	0.6432	0.6071	0.6068	0.5923	0.6398	0.6103	0.6518
59	0.6132	0.6140	0.6148	0.6327	0.6810	0.5832	0.6068
6	0.6425	0.6196	0.5975	0.6327	0.6597	0.6309	0.5904

60	0.6279	0.6419	0.6261	0.6180	0.6822	0.5846	0.6501
61	0.6330	0.6469	0.6177	0.5612	0.6455	0.6046	0.6292
62	0.6341	0.6682	0.6198	0.6151	0.6791	0.6309	0.6312
63	0.6292	0.6470	0.6156	0.5931	0.6747	0.5779	0.5834
64	0.6260	0.6449	0.5609	0.6517	0.6712	0.6374	0.6391
65	0.6037	0.6453	0.5989	0.6083	0.6791	0.5754	0.5962
66	0.6189	0.6617	0.5847	0.6349	0.6815	0.6052	0.6040
67	0.5628	0.6649	0.6343	0.6168	0.6598	0.6228	0.6588
68	0.6249	0.6135	0.6693	0.6152	0.6474	0.5975	0.6115
69	0.6282	0.6598	0.5795	0.5778	0.6617	0.5962	0.6539
7	0.6166	0.6218	0.6375	0.5965	0.6585	0.6138	0.6565
70	0.5988	0.6409	0.6352	0.6213	0.6746	0.5835	0.6314
71	0.6393	0.6220	0.6437	0.5927	0.6685	0.6372	0.6168
72	0.6069	0.6640	0.5612	0.5952	0.6492	0.6142	0.5898
73	0.5812	0.6473	0.6280	0.6428	0.6693	0.5924	0.6067
74	0.6308	0.6535	0.6337	0.6392	0.6902	0.5959	0.6583
75	0.6172	0.6180	0.6137	0.5636	0.6762	0.5829	0.5817
76	0.6568	0.6311	0.5507	0.5910	0.6898	0.5730	0.5843
77	0.6214	0.6534	0.5892	0.6245	0.6585	0.5891	0.5680
78	0.6358	0.6486	0.5668	0.6471	0.6633	0.6002	0.6029
79	0.6245	0.6629	0.5945	0.6270	0.6707	0.6230	0.6493
8	0.6421	0.5883	0.6229	0.6116	0.7047	0.6184	0.6485
80	0.5882	0.6519	0.6033	0.6506	0.6688	0.6132	0.6037
81	0.6373	0.6325	0.6295	0.5971	0.6519	0.6240	0.6609
82	0.5891	0.6566	0.5800	0.6172	0.6781	0.6329	0.5844
83	0.5912	0.6277	0.6170	0.5749	0.6855	0.6631	0.6276
84	0.6488	0.6214	0.5955	0.5702	0.6351	0.6400	0.6105
85	0.6036	0.6283	0.6156	0.5835	0.6737	0.6000	0.6134
86	0.5878	0.6742	0.6680	0.6260	0.6542	0.6204	0.6410
87	0.5878	0.6456	0.6213	0.5479	0.6729	0.5802	0.6116
88	0.6133	0.6104	0.5718	0.5908	0.6774	0.5695	0.6213

89	0.6347	0.6325	0.5970	0.6202	0.6970	0.5627	0.5653
9	0.6549	0.6421	0.6100	0.6106	0.6485	0.6421	0.6277
90	0.6342	0.6517	0.6054	0.6632	0.6746	0.6263	0.6044
91	0.6341	0.6407	0.5863	0.6036	0.6823	0.5974	0.6164
92	0.6273	0.5746	0.5628	0.5924	0.6944	0.6388	0.6068
93	0.6116	0.6148	0.6055	0.6106	0.6630	0.6140	0.6290
94	0.6245	0.6576	0.5776	0.6356	0.6744	0.5987	0.6151
95	0.6199	0.6469	0.6231	0.6405	0.6680	0.5924	0.6521
96	0.6518	0.6597	0.5751	0.6552	0.6563	0.6214	0.6274
97	0.5939	0.6517	0.6053	0.6191	0.6826	0.6116	0.6113
98	0.6121	0.6196	0.5785	0.6212	0.6950	0.6065	0.6298
99	0.6534	0.6524	0.6068	0.6292	0.6593	0.6037	0.6180
**Min**	0.5571	0.5746	0.5330	0.5370	0.6351	0.5495	0.5653
**Q1**	0.6111	0.6304	0.5881	0.5913	0.6643	0.5948	0.6026
**Q2**	0.6245	0.6437	0.6068	0.6161	0.6731	0.6134	0.6166
**Average**	0.6200	0.6424	0.6059	0.6098	0.6727	0.6104	0.6161
**Std. dev.**	0.0218	0.0212	0.0273	0.0277	0.0136	0.0233	0.0230
**Q3**	0.6341	0.6567	0.6239	0.6282	0.6817	0.6290	0.6316
**Max**	0.6568	0.6907	0.6693	0.6632	0.7047	0.6631	0.6609
**Skew**	-0.7234	-0.3496	-0.2840	-0.4989	-0.1472	-0.2050	-0.0599
**Kurtosis**	0.1738	0.4097	-0.1990	-0.3143	0.1528	-0.5068	-0.7172

Table S4. Peptide sequences that are contained both in the AVPDiscover reduced test set and in the Stack-AVP training set.

Sequence	AVPDiscover ID	Stack-AVP ID
YPSKPDNPGEDAPAEDMARYYSALRHYINLITRQRY	Sequence_0_1_testing	Positive_1156
YQEPVLGPVRGPFPIIV	Sequence_2_1_testing	Positive_62
WKSESVCTPGCVTGLLQTCFLQTITCNCKISK	Sequence_5_1_testing	Positive_3732
VTSWSLCTPGCTSPGGGSNCSFCC	Sequence_6_1_testing	Positive_4452
VLSKSLCTPGCITGPLQTCYLCFPTFAKC	Sequence_10_1_testing	Positive_147
VKSTGRADDDLAVKTKYLPP	Sequence_11_1_testing	Positive_575
VKLIQIRIWIQYVTVLQMFSMKTKQ	Sequence_13_1_testing	Positive_562
VIVFVASVAAEMMQHVYCAASKKC	Sequence_16_1_testing	Positive_2428
VCGETCFGGTCNTPGCSCTWPICTRDGLP	Sequence_17_1_testing	Positive_3089
VDKPPYLPRPRPPRRIYNR	Sequence_18_1_testing	Positive_3216
VDKPPYLPRPPPPRRIYNNR	Sequence_19_1_testing	Positive_3733
SVLSTITDMAKAAGRAALNAITGLVNQ	Sequence_20_1_testing	Positive_2869
SVLGTVKDLLIGAGKSAAQSVLTTLSCKLSNSC	Sequence_24_1_testing	Positive_3175
SWFSRTVHNVGNAVRKGIHAGQGVCSGLGL	Sequence_27_1_testing	Positive_3093
SVSCLRNKGVCMPGKCAPKMKQIGTCGMPQVKCCKRK	Sequence_28_1_testing	Positive_2456
SVMGTVKDLLIGAGKSAAQSVLKALSCKLSKDC	Sequence_29_1_testing	Positive_709
SRWPSPGRPRPFPGRPNPIFRPRPCICVRQPCPCDTY	Sequence_30_1_testing	Positive_507
SNDIYFNFQR	Sequence_31_1_testing	Positive_1160
SMISVLKNLGKVGLGFVACKVNKQC	Sequence_32_1_testing	Positive_2953
SSGWVCTLTIECGTVICAC	Sequence_33_1_testing	Positive_1598
SLGSFMKGVGKGLATVGKIVADQFGKLLEAGQG	Sequence_35_1_testing	Positive_43
SLLSLLRKLIT	Sequence_38_1_testing	Positive_2463
SILPTIVSFLSKVF	Sequence_39_1_testing	Positive_970
SIFPAIVSFLSKFL	Sequence_42_1_testing	Positive_2015
SKITDILAKLGKVLAHV	Sequence_44_1_testing	Positive_4568
SILSGNFGVGKKIVCGLSGLC	Sequence_45_1_testing	Positive_628
SIPCGESCVFIPCTVTALLGCSCKSKVCYKN	Sequence_46_1_testing	Positive_3279
SIRDKIKTIAIDLAKSAGTGVLKTLICKLNKSC	Sequence_47_1_testing	Positive_495
SISCGETCTTFNCWIPNCKCNHHDKVCYWN	Sequence_49_1_testing	Positive_3864

SFGLCRLRRGSCAHGRCRFPSIPIGRCSRFVQCCRRVW	Sequence_50_1_testing	Positive_1075
SFLSTFKELAINAAKNAGQSLLHTLSCKLDKTC	Sequence_52_1_testing	Positive_3464
SFHVFPPWMCKSLKKC	Sequence_55_1_testing	Positive_1276
SFLTTFKDLAIKAAKSAGQSVLSTLSCKLSNTC	Sequence_56_1_testing	Positive_2292
SALVGCWTKSYPPNPCFGRG	Sequence_57_1_testing	Positive_19
RYCERSSGTWSGVCGNTDKCSSQCQRLEGAAHGSCNYVFPAHKCICYYPC	Sequence_61_1_testing	Positive_1870
SALVGCGTKSYPPKPCFGR	Sequence_62_1_testing	Positive_1320
SALVGCWTKSWPPKPCFGRG	Sequence_63_1_testing	Positive_1979
SAISCGETCFKFKCYTPRCSCSYPVCK	Sequence_64_1_testing	Positive_2756
RTCMIKKEGWGKCLIDTTCAHSCKNRGYIGGDCKGMTRTCYCLVNC	Sequence_66_1_testing	Positive_2074
RTCRCRFGRCFRRESYSGSCNINGRIFSLCCR	Sequence_67_1_testing	Positive_1992
RRSRRGRGGGRRGGSGGRGGRGGGGRSGAGSSIAGVGSRGGGGGRHYA	Sequence_68_1_testing	Positive_2861
RTCESQSHRFKGTCVRQSNCAAVCQTEGFHGGNCRGFRRRCFCTKHC	Sequence_69_1_testing	Positive_333
RRICRCRIGRCLGLEVYFGVCFLHGRLARRCCR	Sequence_72_1_testing	Positive_1022
RRLHPQHQRFPRERPWPKPLSLPLPRPGPRPWPKPL	Sequence_75_1_testing	Positive_2449
RPDKPRPYLPRPRPPRPVR	Sequence_78_1_testing	Positive_3192
RKFHEKHHSHREFPFYGDYGSNYLYDN	Sequence_86_1_testing	Positive_1886
RIKRFWPVVIRTVVAGYNLYRAIKKK	Sequence_88_1_testing	Positive_4453
RHCESLSHRFKGPCTRDSNCASVCETERFSGGNCHGFRRRCFCTKPC	Sequence_90_1_testing	Positive_2362
RCVCRRGVCRCVCTRGFC	Sequence_91_1_testing	Positive_2148
QWGYNSYGYGNYGGYGGYPMYGGYGMNGGYGGGGLLGMFLGKKK	Sequence_92_1_testing	Positive_1695
QWGYGGYGRGYGGYGGYGRGYGGYGRGYGGYGRGMWGRPYGGYGWGK	Sequence_93_1_testing	Positive_2859
RFRPPIRRPPIRPPFYPPFRPPIRPPIFPPIRPPFRPPLGPFP	Sequence_94_1_testing	Positive_588
QWGYNSYGGYNSYGNYGGYGGGYNNGYGVNANLGVGGRGG	Sequence_95_1_testing	Positive_3374
RCVCTRGFCRCVCTRGFC	Sequence_96_1_testing	Positive_3012
RCVCTRGFCRCICLLGIC	Sequence_97_1_testing	Positive_4225
RFRPPIRRPPIRPPFNPPFRPPVRPPFRPPFRPPFRPPIGPFP	Sequence_100_1_testing	Positive_3128
RFGRFLRKIRRFRPKVTITIQGSARFG	Sequence_101_1_testing	Positive_1805
QVVRNPQSCRWNMGVCIPISCPGNMRQIGTCFGPRVPCCRRW	Sequence_103_1_testing	Positive_2743
RCVCTRGFCRCFCRRGVC	Sequence_104_1_testing	Positive_3321
QWGYGPYGGYGGGYPGMYGGYGMRPYGMYGGYGMGMYRPGLLGMLIGK	Sequence_105_1_testing	Positive_1672

QLPFVAGVACEMCQCVYCAASKKC	Sequence_107_1_testing	Positive_2055
QFTNVSCTTSKECWSVCQRLHNTSRGKCMNKKCRCYS	Sequence_109_1_testing	Positive_3769
QICKAPSQTFPGLCFMDSSCRKYCIKEKFTGGHCSKLQRKCLCTKPC	Sequence_110_1_testing	Positive_2506
QGVRNHVTCRINRGFCVPIRCPGRTRQIGTCFGPRIKCCRSW	Sequence_111_1_testing	Positive_1078
QGVRSYLSCWGNRGICLLNRCPGRMRQIGTCLAPRVKCCR	Sequence_112_1_testing	Positive_404
QAFKTFTPDWNKIRNDAKRMQDNLEQMKKRFNLNL	Sequence_113_1_testing	Positive_4371
PKRKSATKGDEPA	Sequence_114_1_testing	Positive_1460
NRLSCHRNKGVCVPSRCPRHMRQIGTCRGPPVKCCRKK	Sequence_116_1_testing	Positive_2492
NSKRACYREGGECLQRCIGLFHKIGTCNFRFKCCKFQ	Sequence_117_1_testing	Positive_2943
NTCENLAGSYKGVCFGGCDRHCRTQEGAISGRCRDDFRCWCTKNC	Sequence_118_1_testing	Positive_4426
NALSSPRNKCDRASSCFG	Sequence_120_1_testing	Positive_3299
NILNTIINLAKKIL	Sequence_121_1_testing	Positive_1799
NGVYCNKQKCWVDWSRARSEIIDRGVKAYVNGFTKVLGGIGGR	Sequence_122_1_testing	Positive_4469
MGAIAKLVAKFGWPIVKKYYKQIMQFIGEGWAINKIIDWIKKHI	Sequence_138_1_testing	Positive_3429
MFFSSKKCKTVSKTFRGPCVRNAN	Sequence_139_1_testing	Positive_1172
LQDAALGWGRRCPRCPRCPRCSWCPRCPTCPRCNCNPK	Sequence_142_1_testing	Positive_3817
LSPNLLKSLL	Sequence_144_1_testing	Positive_4406
LSKKLICYCRIRGCKRRERVFGTCRNLFLTFVFCCS	Sequence_145_1_testing	Positive_72
LQDAALGWGRRCPRCPRCPNCRRCPRCPTCPSCNCNPK	Sequence_146_1_testing	Positive_2788
LRPAVIRPKGK	Sequence_147_1_testing	Positive_1205
LRPAFIRPKGK	Sequence_148_1_testing	Positive_1144
LLSLVPHAINAVSAIAKHF	Sequence_151_1_testing	Positive_3839
KYYGNGVTCGKHSCSVDWGKATTCIINNGAMAWATGGHQGTHKC	Sequence_154_1_testing	Positive_4129
KYYGNGVHCGKHSCTVDWGTAIGNIGNNAAANWATGGNAGWNK	Sequence_155_1_testing	Positive_2518
LFCKRGTCHFGRCPSHLIKVGSCFGFRSCCKWPWDA	Sequence_156_1_testing	Positive_3541
KYYGNGVTCGKHSCSVNWGQAFSCSVSHLANFGHGKC	Sequence_159_1_testing	Positive_4535
KYYGNGLSCSKKGCTVNWGQAFSCGVNRVATAGHHKC	Sequence_160_1_testing	Positive_1671
KVNANAIKKGGKAIGKGFKVISAASTAHDVYEHIKNRRH	Sequence_161_1_testing	Positive_954
KSCCRSTTARNIYNGCRVPGTARPVCAKKSGCKIQEAKKCEPPYD	Sequence_163_1_testing	Positive_4566
KSCCPTTTARNIYNTCRFGGGSRPVCAKLSGCKIISGTKCDSGWNH	Sequence_165_1_testing	Positive_2731
KSWSLCTPGCARTGSFNSYCC	Sequence_166_1_testing	Positive_2316

KSYGNGVHCNKKKCWVDWGSAISTIGNNSAANWATGGAAGWKS	Sequence_167_1_testing	Positive_1296
KVCRQRSAQFKGPCVSDKNCAQVCLQEQWQQQNCDQPFRRCKCIRQC	Sequence_168_1_testing	Positive_1685
KTCENLVDTYRGPCFTTGSCDDHCKNKEHLLSGRCRDDVRCWCTRNC	Sequence_172_1_testing	Positive_3356
KRFKKFFKKLKNSVKKRAKKFFKKPKVIGVTFPF	Sequence_174_1_testing	Positive_717
KPAWCWYTLAMCGAGYDSGTCDYMYSHCFGIKHHSSGSSSYHC	Sequence_177_1_testing	Positive_1264
KQEGRDHDKSKGHFHMIVIHHKGGQAHHG	Sequence_178_1_testing	Positive_2671
KLCERSSGTWSGVCGNNNACKNQCINLEGARHGSCNYVFPYHRCICYFPC	Sequence_181_1_testing	Positive_1194
KKLLKWLKKLL	Sequence_183_1_testing	Positive_1228
KICRRRSAGFKGPCMSNKNCAQVCQQEQWQQQNCDQPFRRCKCIRQC	Sequence_185_1_testing	Positive_319
KICERASGTWKGICIHSNDCNNQCVKWENAGSGSCHYQFPNYMCFCYFNC	Sequence_187_1_testing	Positive_2159
KGRGKQGGKVRAKAKTRSS	Sequence_188_1_testing	Positive_3030
KDCKTESNTFPGICITKPPCRKACIKEKFTDGHCSKILRRCLCTKPC	Sequence_189_1_testing	Positive_2378
IRNSLTCRFNFGICLPKRCPGRMRQIGTCF	Sequence_195_1_testing	Positive_893
INWLKLGKKMMSAL	Sequence_196_1_testing	Positive_3186
INWLKLGKKILGAL	Sequence_198_1_testing	Positive_1497
ILPLLLGKVVCAITKKC	Sequence_199_1_testing	Positive_4195
INLKAIAALAKKLL	Sequence_203_1_testing	Positive_3072
ILPFLAGLFSKIL	Sequence_206_1_testing	Positive_2502
ILPIIGKILSTIF	Sequence_207_1_testing	Positive_898
INWKKIFEKVKNLV	Sequence_208_1_testing	Positive_641
ILPFVAGVAAEMMEHVYCAASKKC	Sequence_209_1_testing	Positive_2304
INIKDILAKLVKVLGHV	Sequence_210_1_testing	Positive_4475
IKIMDILAKLGKVLAHV	Sequence_213_1_testing	Positive_2542
ILGKLLSTAAGLLSNL	Sequence_215_1_testing	Positive_2288
IKIPPIVKDTLKKVAKGVLSTIAGALST	Sequence_218_1_testing	Positive_1576
IKIPAVVKDTLKKVAKGVLSAVAGALTQ	Sequence_219_1_testing	Positive_1167
ILGPVLSMVGSALGGLIKKI	Sequence_220_1_testing	Positive_838
ILGPVISKIGGVLGGLLKNL	Sequence_221_1_testing	Positive_2841
IDWLKLGKMVIDAL	Sequence_222_1_testing	Positive_3271
IIGHLIKTALGFLGL	Sequence_224_1_testing	Positive_4587
IDWLKLGKMVMDVL	Sequence_228_1_testing	Positive_134

IGVIKLSLCEEERNADEEKRRDDPDEMDVEVEKR	Sequence_229_1_testing	Positive_660
IIGPVLGMVGSALGGLLKKI	Sequence_232_1_testing	Positive_398
IIGAIAAALPHVINAIKNTF	Sequence_233_1_testing	Positive_1039
IFGAIWNGIKSLF	Sequence_234_1_testing	Positive_375
HFLGGTLVNLAKKIL	Sequence_236_1_testing	Positive_3051
GWLRKAAKSVGKFYYKHKYYIKAAWKIGRHAL	Sequence_238_1_testing	Positive_1626
GWFKKAWRKVKNAGRRVLKGVGIHYGVGLI	Sequence_239_1_testing	Positive_2513
GVVDILKGAGKDLLAHALSKLSEKV	Sequence_240_1_testing	Positive_2268
GWKKWFNRAKKVGKTVGGLAVDHYL	Sequence_241_1_testing	Positive_2026
GWRLLLKKAEVKTVGKLALKHYL	Sequence_242_1_testing	Positive_2780
GVVTDLLKTAGKLLGNLFGSLSG	Sequence_243_1_testing	Positive_4024
GWMSKIASGIGTFLSGVQQG	Sequence_244_1_testing	Positive_3377
GVVDILKGAAKDIAGHLASKVMNKL	Sequence_245_1_testing	Positive_1372
GTPCGESCVYIPCFTAVVGCTCKDKVCYLN	Sequence_248_1_testing	Positive_4295
GVLGAVKDLLIGAGKSAAQSVLKTLSCKLSNDC	Sequence_251_1_testing	Positive_3707
GVIKSVLKGVAKTVALGML	Sequence_254_1_testing	Positive_40
GTFPCGESCVWIPCISKVIGCACKSKVCYKN	Sequence_255_1_testing	Positive_14
GVFTLIKGATQLIGKTLGKELGKTGLELMACKITEQC	Sequence_257_1_testing	Positive_2829
GTRCGETCFVLPCWSAKFGCYCQKGFCYRN	Sequence_260_1_testing	Positive_3273
GVFSFLKTGAKLLGSTLLKMAGKAGAEHLACKATNQC	Sequence_265_1_testing	Positive_2092
GSVPCGESCVYIPCFTGIAGCSCKSKVCYYN	Sequence_267_1_testing	Positive_2443
GTPCAESCVYLPCFTGVIGCTCKDKVCYLN	Sequence_269_1_testing	Positive_3965
GVLATVKNLLIGTGDGAAQSVLKTLSCKLSNDC	Sequence_270_1_testing	Positive_3728
GNIPCGESCIFFPCFNPGCSCKDNLCYYN	Sequence_277_1_testing	Positive_1281
GMFSVLKNLGKVGLGFVACKINKQC	Sequence_278_1_testing	Positive_678
GLWSKIKEAAKTAGKMAMGFVNDMV	Sequence_279_1_testing	Positive_1930
GLWQFIKDKLKDAATGLVTGIQS	Sequence_280_1_testing	Positive_1056
GLWSTIKNVGKEAAIAAGKAVLGSLGEQ	Sequence_282_1_testing	Positive_4028
GLWSKIKTAGKEAAKAAAKAAGKAALNAVSEAI	Sequence_285_1_testing	Positive_1210
GMWSKIKNAGKAAKAAAKAAGKAALGAVSEAM	Sequence_286_1_testing	Positive_3373
GLVSDLLSTVTGLLGNLGGGGLKKI	Sequence_290_1_testing	Positive_3983

GLWDSIKNFGKTIALNVMDKIKCKIGGGCPP	Sequence_291_1_testing	Positive_3402
GLRSKIKEAAKTAGKMALGFVNDMA	Sequence_297_1_testing	Positive_2968
GLWDTIKQAGKKIFLSVLDKIRCKVAGGG	Sequence_303_1_testing	Positive_2666
GLVRKGGEKFGEKLRKIGQKIKEFFQKLALEIEQ	Sequence_304_1_testing	Positive_4495
GLVTSLIKGAGKLLGGLFGSVTG	Sequence_306_1_testing	Positive_1149
GLVTGLLKTAGKLLGDLFGSLTG	Sequence_309_1_testing	Positive_3098
GLPCGETTCFTGKCYTPGCSCSYPICKKIN	Sequence_311_1_testing	Positive_2973
GLVSGLLNTAGGLLGDLLGSLGSLSGGES	Sequence_312_1_testing	Positive_2536
GLPVCGETCVGGTCNTPGCSCSRPVCTAN	Sequence_313_1_testing	Positive_2391
GLLLDTLKGAAKDIAGIALEKLKCKITGCKP	Sequence_315_1_testing	Positive_914
GLMDVFKGAAKNLLASALDKIRCKVTKC	Sequence_317_1_testing	Positive_818
GLLSVLKGVLKTTGKHIFKNVGGSLLDQAKCKISGQC	Sequence_318_1_testing	Positive_2093
GLLSGILNSAGGLLGNLIGSLSN	Sequence_319_1_testing	Positive_4296
GLLDTLKGAAKNVVGSLASKVMEKL	Sequence_320_1_testing	Positive_966
GLLDTFKNMAINAAHGAGVSVLNALSCKLKKTC	Sequence_321_1_testing	Positive_2914
GLMDSLKGLAATAGKTVLQGLLKTASCKLEKTC	Sequence_322_1_testing	Positive_2081
GLLGGLLGPLLGGGGGGGGLL	Sequence_325_1_testing	Positive_1246
GLMSTLKDFGKTAAKEIAQSLLSTASCKLAKTC	Sequence_326_1_testing	Positive_843
GLLSGILGAGKHIVCGLTGCAKA	Sequence_327_1_testing	Positive_851
GLLDTIKNMALNAAKSAGVSVLNSLSCKLSKTC	Sequence_331_1_testing	Positive_661
GLMSLFRGVLKTAGKHIFKNVGGSLLDQAKCKITGEC	Sequence_334_1_testing	Positive_824
GLLSSFKGVAKGVAKDLAGKLLEKLKCKITGC	Sequence_335_1_testing	Positive_1349
GLLGAMFKVASKVLPHVVPAITEHF	Sequence_336_1_testing	Positive_2565
GLLSGILGAGKHIVCGLSGLK	Sequence_337_1_testing	Positive_3173
GLLDTFKNLAINAAESAGVSVLNSLSCKLSKTC	Sequence_338_1_testing	Positive_2740
GLMSVLGHAVGNVLGGLFKS	Sequence_341_1_testing	Positive_726
GLLSGVLGVGKKVLCGLSGLC	Sequence_342_1_testing	Positive_3676
GLFSKFAGKGIKNFLIKGVKHIGKEVGMDVIRVGIDVAGCKIKGVC	Sequence_343_1_testing	Positive_4188
GLLDFAKHVIGIASKL	Sequence_347_1_testing	Positive_3329
GLFTLIKGAAKLIGKTTAKEAGKTGKLEMACKITNQC	Sequence_349_1_testing	Positive_3771
GLFLNTVKDVAKDVAKDVAGKLLESLKCKITGCKS	Sequence_351_1_testing	Positive_3894

GLFSILKGVGKIALKGLAKNMGKMGLDLVSCKISKEC	Sequence_353_1_testing	Positive_4317
GLFSAFKKVGKNVLKNVAGSLMDNLKCKVSGEC	Sequence_355_1_testing	Positive_636
GLISGILGVGKMLVCGLSGLC	Sequence_357_1_testing	Positive_935
GLFTLIKGAYKLDAPTVACN	Sequence_359_1_testing	Positive_21
GLFKVLGSVAKHLLPHVVPVIAEKL	Sequence_361_1_testing	Positive_2062
GLGSFFKNAIKIAGKVGSTIGKVADAIGNKE	Sequence_363_1_testing	Positive_1897
GLFSKLNKKKIKSGLIKIIKTAGKEAGLEALRTGIDVIGCKIKGEC	Sequence_366_1_testing	Positive_3719
GLKEIFKAGLGSLVKGIAAHVAS	Sequence_367_1_testing	Positive_1184
GLFPKINKKKAKTGVFNIIKTVGKEAGMDLIRTGIDTIGCKIKGEC	Sequence_369_1_testing	Positive_4528
GLFGKSSVWGRKYYVDLAGCAKA	Sequence_370_1_testing	Positive_2458
GLFTKFAGKGIKDLIFKGVKHIGKEVGMDVIRVGIDVAGCKIKGVC	Sequence_371_1_testing	Positive_75
GLASFLGKALKAGLKIGSHLLGGAPQQ	Sequence_372_1_testing	Positive_2803
GKPRPYSPRPTSHPRPIRV	Sequence_373_1_testing	Positive_2969
GLFDSIKNVAKNVAAGLLDKLKCKITGC	Sequence_374_1_testing	Positive_4280
GKFSGFAKILKSIAKFFKGVGKVRKGFKEASDLDKNQ	Sequence_375_1_testing	Positive_2147
GIMDTVKNAAKDLAGQLLDKLKCRITGC	Sequence_376_1_testing	Positive_1222
GIPCGESCVFIPCISGVIGCSCKSKVCYRN	Sequence_377_1_testing	Positive_2777
GIMDSVKNVAKNIAGQLLDKLKCKITGC	Sequence_378_1_testing	Positive_2670
GIPCGESCVYIIPCTVTALAQCKCKSKVCYN	Sequence_381_1_testing	Positive_3973
GIPCGESCVWIPCLTSAIGCSCKSKVCYKD	Sequence_384_1_testing	Positive_1977
GIMDSVKNAAKNLAGQLLDTIKCKITAC	Sequence_386_1_testing	Positive_531
GILSNVLGMGKKIVCGLSGLC	Sequence_388_1_testing	Positive_1535
GILSSFKGVAKGVAKNLAGKLLDELKCKITGC	Sequence_389_1_testing	Positive_1404
GIMDSVKGVAKNLAAKLLEKLKCKITGC	Sequence_391_1_testing	Positive_1606
GIPCGESCVWIPGISAAIGCSCKNKVCYRN	Sequence_392_1_testing	Positive_3961
GIMDSIKGLGKNLAGQLLDKLKCKITGC	Sequence_397_1_testing	Positive_1736
GIPCGESCVWIPCLTSAVGCPCKSKVCYRN	Sequence_398_1_testing	Positive_1202
GIGTKFLGGVKTALKGALKELAFTYVN	Sequence_404_1_testing	Positive_3659
GILGTVFKAGKGIVCGLTGLC	Sequence_405_1_testing	Positive_2312
GILDTFKGVAKGVAKDLAVHMLENLKCKMTGC	Sequence_406_1_testing	Positive_2820
GIGSALAKAAKLVAGIV	Sequence_407_1_testing	Positive_3203

GILSGILGAGKSLVCGLSGLC	Sequence_408_1_testing	Positive_1355
GILDTIKNAAKTVAVGLLEKIKCKMTGC	Sequence_412_1_testing	Positive_582
GIKDWIKGAAKTLIKTVASHIANQ	Sequence_414_1_testing	Positive_3600
GIGTKILGGVKAALKGALKELASTYVN	Sequence_415_1_testing	Positive_86
GIFSKISGKAIKNLFIKGAKNVGKRVGMDVVRTGMDVVGCKIKGEC	Sequence_420_1_testing	Positive_4128
GIGGALLSVGKLALKGLANVLADKFAN	Sequence_422_1_testing	Positive_3963
GIGGALLSAGKSALKGLAKGLAEHF	Sequence_423_1_testing	Positive_897
GIFSKFAGKGIKNLLVKGVKNIGKEVGMDVIRTGIDIAGCKIKGEC	Sequence_424_1_testing	Positive_501
GIGGKILGGLRTALKGAAKELAATYLH	Sequence_427_1_testing	Positive_4202
GIGALSAKGALKGLAKGLAEHFAN	Sequence_432_1_testing	Positive_111
GIFSLIKGAAKLITKTVAKEAGKTGLELMACKVTNQC	Sequence_433_1_testing	Positive_957
GIFSLIKTAAKFVGKNLLKQAGKAGVEHLACKANNQC	Sequence_434_1_testing	Positive_662
GIFSKINKKKAKTGLFNIIKTVGKEAGMDVIRAGIDTISCKIKGEC	Sequence_439_1_testing	Positive_205
GIFSALAAGVKLLGNTLFKMAGKAGAEHLACKATNQC	Sequence_442_1_testing	Positive_1070
GIFGKILGVGKKVLCGLSGWC	Sequence_443_1_testing	Positive_583
GIFGKILGVGKKTLCELSGMC	Sequence_444_1_testing	Positive_2774
GIFNVFKGALKTAGKHVAGSLLNQLKCKVSGEC	Sequence_445_1_testing	Positive_1943
GICRCLCRRGVCRCICVL	Sequence_446_1_testing	Positive_4212
GICRCICTRGFCRCICVL	Sequence_447_1_testing	Positive_3506
GFMATAKNVAKNMDVTLLDNLKCKITKAC	Sequence_451_1_testing	Positive_3300
GFMDTAKNVAKNVAVTLIDKLRCKVTGGC	Sequence_452_1_testing	Positive_1925
GGKPDLRPCHPPCHYIPRPKPR	Sequence_454_1_testing	Positive_4001
GFMDTAKNVAKNVAVTLIDNLKCKITKAC	Sequence_457_1_testing	Positive_51
GFRDVLKGAAKQFVKTVAGHIANI	Sequence_460_1_testing	Positive_3219
GFGSFLGKALKAALKIGANVLGGAPQQ	Sequence_462_1_testing	Positive_3716
GFGCPLNQGACHRHCRSIRRRGGYCAGFFKQTCCYRN	Sequence_465_1_testing	Positive_2139
GFGSLLGKALRLGANVL	Sequence_470_1_testing	Positive_3524
GFGCPWNRYQCHSHCRSIGRLGGYCAGSLRLTCTCYRS	Sequence_471_1_testing	Positive_4506
GFGCPNDYPCHRHCKSIPGRAGGYCGGAHRLRCTCYR	Sequence_474_1_testing	Positive_4098
GFGSLLGKALKIGTNLL	Sequence_475_1_testing	Positive_1041
GFKDWIKSAAKKLIKTVASNIANQ	Sequence_476_1_testing	Positive_2255

GFKDWIKGAAKKLIKTVASSIANE	Sequence_478_1_testing	Positive_2768
GFGCPNNYACHQHCKSIRGYCGGYCAGWFRLRCTCYRCG	Sequence_479_1_testing	Positive_4018
GFGCPNNYQCHRHCKSIPGRCGGYCGGWHRLPCTCYRCG	Sequence_480_1_testing	Positive_1622
GEIPCGESCVYLPCFLPNCYCRNHVCYLN	Sequence_481_1_testing	Positive_1948
FTSKKSMLLFFFLGTISLSLCQ	Sequence_482_1_testing	Positive_1346
FVDLKKIANIINSIF	Sequence_484_1_testing	Positive_2052
FMGSALRIAAKVLPAALCQIFKKC	Sequence_487_1_testing	Positive_2240
FLSLLPSLVSGAVSLVKKL	Sequence_488_1_testing	Positive_4340
FLSLIPTAINAVSALAKHF	Sequence_489_1_testing	Positive_1343
FLSLIPHAINAVSTLVHHS	Sequence_491_1_testing	Positive_1476
FLPLLAGVVANFLPQIICKIARKC	Sequence_493_1_testing	Positive_322
FLPVLARLAVKFLPSIVCAATKKC	Sequence_496_1_testing	Positive_41
FLPVIAGVAAKFLPKIFCAITKKC	Sequence_497_1_testing	Positive_1374
FLPILGNLLSGLL	Sequence_498_1_testing	Positive_622
FLPVIAGLLSKLF	Sequence_499_1_testing	Positive_663
FLPLIASVAANLAPKIICKITKTC	Sequence_501_1_testing	Positive_2992
FLSAITSILGKFF	Sequence_502_1_testing	Positive_1625
FLPIVAKLLSGLL	Sequence_503_1_testing	Positive_1617
FLPLLGNLLRGLL	Sequence_504_1_testing	Positive_769
FLPLVLGALSGILPKIL	Sequence_505_1_testing	Positive_4131
FLPIVGRLISGLL	Sequence_506_1_testing	Positive_221
FLSLIPHAINAVSAIAKHF	Sequence_509_1_testing	Positive_4526
FLPIITNLLGKLL	Sequence_510_1_testing	Positive_201
FLPIIASVAAKLIPSIVCRITKKC	Sequence_511_1_testing	Positive_3004
FLPAVLLVATHVLPTVFCAITRKC	Sequence_512_1_testing	Positive_647
FLGSLIGAAIPAIKQLLGLKK	Sequence_513_1_testing	Positive_2757
FLGLLPSIVSGAVSLVKKL	Sequence_515_1_testing	Positive_3957
FLPIALKALGSIFPKIL	Sequence_516_1_testing	Positive_355
FLPIAGKLLSGLSGLL	Sequence_517_1_testing	Positive_3062
FLGPIIKIATGILPTAICKFLKKC	Sequence_518_1_testing	Positive_3315
FLPFLKSILGKIL	Sequence_519_1_testing	Positive_4497

FLPFFASLLGKLL	Sequence_520_1_testing	Positive_2328
FLPIIGQLLSGLL	Sequence_521_1_testing	Positive_1003
FLPILAGLAANILPKVFCSITKKC	Sequence_522_1_testing	Positive_280
FLFRVASKVFPALIGKFKKK	Sequence_523_1_testing	Positive_4279
FLGGLMKIIPAAFCAVTKKC	Sequence_524_1_testing	Positive_2329
FFPLVLGALGSILPKIF	Sequence_525_1_testing	Positive_3454
FIHHIIGGLFSAGKAIHRLIRRRRR	Sequence_526_1_testing	Positive_2187
FFPIVGKLLFGLSGLL	Sequence_527_1_testing	Positive_3140
FFPIVGKLLSGLL	Sequence_530_1_testing	Positive_3032
FFPIIAGMAAKLIPSLFCKITKKC	Sequence_531_1_testing	Positive_1127
FFPMLAGVAARVVPKVICLITKKC	Sequence_533_1_testing	Positive_136
FALGAVTKRLPSLFCLITRKC	Sequence_538_1_testing	Positive_302
FASLLGKALKALAKQ	Sequence_539_1_testing	Positive_3760
FDNPFGCPADEGKCFDHCNNKAYDIGYCGGSYRATCVCYRK	Sequence_540_1_testing	Positive_4148
FFGTALKIAANVLPTAICKILKKC	Sequence_541_1_testing	Positive_402
FFGSVLKVAAKVLPAALCQIFKKC	Sequence_542_1_testing	Positive_421
EPFKLSLHL	Sequence_543_1_testing	Positive_868
EPHPNEFVGLM	Sequence_544_1_testing	Positive_3714
EPNPDEFFGLM	Sequence_545_1_testing	Positive_986
ETCASRCPRPCNAGLCCSIYGYCGSGNAYCGAGNCRCQCRG	Sequence_547_1_testing	Positive_3399
EPNPDEFVGLM	Sequence_548_1_testing	Positive_1442
ELCEKASQTWSGTCGKTKHCDDQCKSWEGAAHGACHVRDGKHMCFCYFNC	Sequence_549_1_testing	Positive_3178
DSHAKRHHGYKRKFHEKHHSHRGYR	Sequence_552_1_testing	Positive_4065
DTVACRIQGNFCRAGACPPTFTISGQCHGGLLNCCAKIPAQ	Sequence_553_1_testing	Positive_34
DTLIGSCVWGATNYTSDCNAECKRRGYKGGHCGSFLNVNCWCE	Sequence_554_1_testing	Positive_1613
DSHEKRHHEHRRKFHEKHHSHRGY	Sequence_556_1_testing	Positive_2311
DFGCGQGMIFMCQRRCMRLYPGSTGFCRGFRCMCDTHIPLRPPFMVG	Sequence_560_1_testing	Positive_4360
DCTRWIIGINGRICRD	Sequence_561_1_testing	Positive_593
DHYICAKKGGTCNFSPCPLFNRIEGTCYSGKAKCCIR	Sequence_562_1_testing	Positive_3088
CSTNTFSLSDYWGNNGAWCTLTHECMAWCK	Sequence_563_1_testing	Positive_977
CVWIPCISGIAGCSCKNKVCYLN	Sequence_564_1_testing	Positive_1397

CRFCCRCCPRMRGCGLCCRF	Sequence_566_1_testing	Positive_3867
CRQSCSFGPLTFVCDGNTK	Sequence_567_1_testing	Positive_4055
CGESCVWIPCISSAVGCSCKNKVCYKNGTP	Sequence_568_1_testing	Positive_933
CGETCLFIPCLTSVFGCSCKNRGCYKI	Sequence_572_1_testing	Positive_3326
CGESCVYIPCLTSAVGCSCKSKVCYRNGIP	Sequence_576_1_testing	Positive_4468
CGETCIWGRCYSENIGCHCGFGICTLN	Sequence_578_1_testing	Positive_4312
CGESCVWIPCISSAIGCSCKSKVCYRNGIP	Sequence_583_1_testing	Positive_4245
CGETCLFIPCIFSVVGCSCSSKVCYRN	Sequence_584_1_testing	Positive_4561
CGETCIYIPCFTEAVGCKCKDKVCYKN	Sequence_585_1_testing	Positive_523
CGETCVVDTRCYTKKCSCAWPVCMRN	Sequence_586_1_testing	Positive_3596
CGETCKVTKRCSGQGCSCLKGRSCYD	Sequence_587_1_testing	Positive_1416
CESCVWIPCISSVVGCSCKSKVCYKNGTLP	Sequence_588_1_testing	Positive_7
CGETCVTGTCYTPGCACDWPVCKRD	Sequence_589_1_testing	Positive_214
CGESCVFIPCISTLLGCSCKNKVCYRNGVIP	Sequence_590_1_testing	Positive_553
CGESCVWIPCTITALAGCKCKSKVCYNSIP	Sequence_591_1_testing	Positive_132
CAESCVYIPCTVTALLGCSCSNRVCYNGIP	Sequence_593_1_testing	Positive_179
AVWKDFLKNIGKAAGKAVLNSVTDMVNE	Sequence_596_1_testing	Positive_1354
CAWYNISCRLGNKGAYCTLTVECMPSCN	Sequence_597_1_testing	Positive_3045
CAETCVVLPCFIVPGCSCKSSVCYFN	Sequence_599_1_testing	Positive_477
CANSCSYGPLTWSCDGNTK	Sequence_600_1_testing	Positive_1505
ATCDLLSGFGVGDSACAAHCIARGNRGGYCNSKKVCVCPI	Sequence_603_1_testing	Positive_3575
ATCDLLSMWNVNHSACAAHCLLLGKSGGRCNDDAVCVCRK	Sequence_604_1_testing	Positive_138
ATCDLFSFRSKWVTPNHAACAAHCLLRGNRGGRCKGTICHCRK	Sequence_605_1_testing	Positive_1209
ASIVKTTIKASKKLCRGFTLTCGCHFTGKK	Sequence_607_1_testing	Positive_961
AVLDFIKAAGKGLVTNIMEKVG	Sequence_609_1_testing	Positive_3122
ATCRKPSMYFSGACFSDTNCQKACNREDWPNGKCLVGFKCECQRPC	Sequence_610_1_testing	Positive_922
ATCDLLSGTGINHSACAAHCLLRGNRGGYCNGKGVCVCRN	Sequence_611_1_testing	Positive_3165
AVLDILKDVGKGLLSHFMEKV	Sequence_612_1_testing	Positive_4108
AQRCGDQARGAKCPNCLCCGKYGFCGSGDAYCGAGSCQSQCRGCR	Sequence_614_1_testing	Positive_352
AMVSS	Sequence_615_1_testing	Positive_464
ALWKTLLKGAGKVFGHVAKQFLGSQGQPES	Sequence_616_1_testing	Positive_1729

AQCGAQGGGATCPGGLCCSQWGWCGSTPKYCGAGCQSNCR	Sequence_617_1_testing	Positive_1480
ALWKDVLKKIGTVALHAGKAALGAVADTISQ	Sequence_619_1_testing	Positive_3153
ALGTLLKGVGSAVATVGKMVADQFGKLLQAGQG	Sequence_620_1_testing	Positive_1299
AICKKPSKFFKGACGRDADCEKACDQENWPGGVCVPFLRCECQRSC	Sequence_623_1_testing	Positive_101
ACYCRIPACFAGERRYGTCFYLGRVWAFCC	Sequence_624_1_testing	Positive_3289
WRKWRKRWWWRKWRKRWW	Sequence_628_1_testing	Positive_1058
TCTLGTCYTAGCSCSWPVCTRNGVPICGE	Sequence_631_1_testing	Positive_82
TCFGGTCNTPGCSCDPWPVCSRNGVPVCGE	Sequence_632_1_testing	Positive_1716
TCVGGTCNTPGCSCSWPVCTRNGLPICGE	Sequence_633_1_testing	Positive_2746
TCFGGTCNTPGCSCETWPVCSRNGLPVCGE	Sequence_635_1_testing	Positive_3874
SCVFIPCISAAIGCSCKNKVCYRNGVIPCGE	Sequence_641_1_testing	Positive_3962
SCVFIPCITSLAGCSCKNKVCYYDGGSVPCGE	Sequence_642_1_testing	Positive_1715
SCVFIPCLTTVAGCSCKNKVCYRNGIPCGE	Sequence_643_1_testing	Positive_2949
SCYVLPCFTVGCTCTSSQCFKNGTACGE	Sequence_644_1_testing	Positive_3633
RLLRRLLRRLLRRLLRRLLR	Sequence_646_1_testing	Positive_4032
KLIWILSKTIPAIKNLFYKI	Sequence_662_1_testing	Positive_278
HTASDAAAAAALTAANAAAAAAASMA	Sequence_663_1_testing	Positive_4031
DCYCRIPACIAGEKKYGTCIYQGKLWAFCC	Sequence_667_1_testing	Positive_4346
DCYCRIPACIAGEAAYGTCIYQGALWAFCC	Sequence_668_1_testing	Positive_1645
CGESCVWIPCVTSIFNCKCKENKVCYHDKIP	Sequence_671_1_testing	Positive_3741
CGESCVFIPCISSVIGCSCSSKVCYRNGIP	Sequence_676_1_testing	Positive_168
CGETCFTGTCYTNGCTCDPWPVCTRNGLPV	Sequence_678_1_testing	Positive_3643
CGESCVFIPCISSVIGCACKSKVCYKNGSIP	Sequence_679_1_testing	Positive_912
CGETCFGGTCNTPGCSCSSWPICTRNGLPV	Sequence_683_1_testing	Positive_4584
CGESCVFIPCISAIIGCSCSSKVCYKNGSIP	Sequence_685_1_testing	Positive_3517
APKAMRLLRRLLRRLLR	Sequence_688_1_testing	Positive_4074

Table S5. Disagreement and double-fault measures calculated between the predictions of the model pairs that were developed from the analyzed distance/threshold pairs. Between parentheses, the adjusted disagreement values are reported.

**Distance/Threshold Pairs**		$\mathbf{D}_{test}^{\pm}$	$\mathbf{DF}_{test}^{\pm}$	$\mathbf{D}_{test}^{+}$	$\mathbf{DF}_{test}^{+}$	$\mathbf{D}_{test}^{-}$	$\mathbf{DF}_{test}^{-}$
Euclidean/26.242	Cosine/0.018	0.1105	0.0345	**0.1130 (0.1073)**	0.0642	0.1102 (0.0770)	0.0311
	Bhattacharyya/1.5158	0.0850	0.0412	0.0813 (0.0569)	0.0707	0.0854 (0.0636)	0.0378
	Canberra/0.6155	0.1070	0.0377	0.0992 (0.0895)	0.0789	0.1079 (0.0733)	0.0330
	Clark/0.4161	0.1008	0.0277	0.1301 (0.0878)	0.0797	0.0975 (0.0958)	0.0217
	Lance-Williams/0.1789	0.1017	0.0372	0.1195 (0.0927)	0.0772	0.0996 (0.0740)	0.0326
	Soergel/0.3035	0.1092	0.0374	0.1309 (0.0927)	0.0772	0.1068 (0.0735)	0.0329
Cosine/0.018	Bhattacharyya/1.5158	0.1282	0.0342	**0.1228 (0.1041)**	0.0472	**0.1288 (0.1174)**	0.0327
	Canberra/0.6155	0.1125	0.0495	0.0984 (0.0829)	0.0764	**0.1141 (0.1127)**	0.0464
	Clark/0.4161	0.1248	0.0302	0.1455 (0.0976)	0.0691	0.1225 (0.0910)	0.0258
	Lance-Williams/0.1789	0.1075	0.0488	0.1089 (0.0764)	0.0797	0.1073 (0.0997)	0.0453
	Soergel/0.3035	0.1274	0.0429	0.1252 (0.0813)	0.0772	**0.1277 (0.1276)**	0.0390
Bhattacharyya/1.5158	Canberra/0.6155	0.1288	0.0352	0.1203 (0.0862)	0.0561	**0.1298 (0.1170)**	0.0329
	Clark/0.4161	0.1153	0.0289	0.1431 (0.0764)	0.0610	0.1122 (0.0921)	0.0253
	Lance-Williams/0.1789	0.1212	0.0359	0.1309 (0.0797)	0.0593	**0.1200 (0.1162)**	0.0332
	Soergel/0.3035	0.1249	0.0381	0.1390 (0.0764)	0.0610	**0.1233 (0.1118)**	0.0355
Canberra/0.6155	Clark/0.4161	0.1230	0.0326	0.1203 (0.0878)	0.0894	0.1233 (0.0904)	0.0261
	Lance-Williams/0.1789	0.0983	0.0548	0.0789 (0.0618)	0.1024	0.1005 (0.0915)	0.0494
	Soergel/0.3035	0.1226	0.0467	0.1146 (0.0862)	0.0902	**0.1235 (0.1222)**	0.0418
Clark/0.4161	Lance-Williams/0.1789	0.1165	0.0327	**0.1341 (0.1187)**	0.0911	0.1145 (0.0906)	0.0260
	Soergel/0.3035	0.1309	0.0295	**0.1504 (0.1463)**	0.0886	0.1287 (0.0971)	0.0227
Lance-Williams/0.1789	Soergel/0.3035	0.1152	0.0472	0.1106 (0.0992)	0.1008	**0.1158 (0.1081)**	0.0411

$D_{test}^{\pm}$, D_{test}^{+}, D_{test}^{-}: disagreement measure on the entire, positive-class, and negative-class AVPDiscover test set, respectively.

$DF_{test}^{\pm}$, DF_{test}^{+}, DF_{test}^{-}: double-fault measure on the entire, positive-class, and negative-class AVPDiscover test set, respectively.

Table S6. Performance metrics achieved on the AVPDiscover test set when combining models trained with graphs derived from different distance thresholds, and whose adjusted disagreement values for the positive class were greater than 0.1. If one of the two models predict AVP, then the final decision is AVP.

**Distance/Threshold Pairs**		**SN**	**SP**	**ACC**	**MCC**
Cosine/0.018	Bhattacharyya/1.5158	0.9358	0.8587	0.8666	0.5791
	Euclidean/26.242	0.9528	0.8385	0.8503	0.56
Clark/0.4161	Lance-Williams/0.1789	0.9089	0.8595	0.8646	0.5634
	Soergel/0.3035	0.9114	0.8486	0.855	0.5483

Table SI7. Performance metrics obtained on the external dataset by the StackAVP and AI4AVP models from the literature, and by the best individual and combined models based on graphs.

Models	SN	SP	ACC	MCC
StackAVP	0.8535	0.7723	0.7748	0.2502
AI4AVP	0.8974	0.5291	0.5404	0.1471
This work (Cosine/0.018)	0.7546	0.7455	0.7458	0.1943
This work (Bhattacharyya/1.5158)	0.7766	0.7167	0.7185	0.1858
This work (Canberra/0.6155)	0.7216	0.7407	0.7401	0.1788
This work (Clark/0.4161)	0.6557	0.7648	0.7614	0.1678
This work (Euclidean/26.242)	0.7326	0.7912	0.7894	0.2163
This work (Lance-Williams/0.1789)	0.6996	0.7547	0.7530	0.1787
This work (Soergel/0.3035)	0.7106	0.7519	0.7506	0.1813
Cosine/0.018, Bhattacharyya/1.5158	0.6667	0.8581	0.8522	0.2480
Bhattacharyya/1.5158, Canberra/0.6155	0.6520	0.8510	0.8449	0.2340
Euclidean/26.242, Cosine/0.018	0.6520	0.8752	0.8684	0.2612
Euclidean/26.242, Clark/0.4161	0.5824	0.8973	0.8877	0.2570
Euclidean/26.242, Cosine/0.018, Bhattacharyya/1.5158	0.6044	0.9016	0.8925	0.2746
